# The PS1 Hairpin of Mcm3 Is Essential for Viability and for DNA Unwinding *In Vitro*

**DOI:** 10.1371/journal.pone.0082177

**Published:** 2013-12-11

**Authors:** Simon K. W. Lam, Xiaoli Ma, Tina L. Sing, Brian H. Shilton, Christopher J. Brandl, Megan J. Davey

**Affiliations:** Department of Biochemistry, Schulich School of Medicine & Dentistry, University of Western Ontario, London, Ontario, Canada; Université de Sherbrooke, Canada

## Abstract

The pre-sensor 1 (PS1) hairpin is found in ring-shaped helicases of the AAA+ family (ATPases associated with a variety of cellular activities) of proteins and is implicated in DNA translocation during DNA unwinding of archaeal mini-chromosome maintenance (MCM) and superfamily 3 viral replicative helicases. To determine whether the PS1 hairpin is required for the function of the eukaryotic replicative helicase, Mcm2-7 (also comprised of AAA+ proteins), we mutated the conserved lysine residue in the putative PS1 hairpin motif in each of the *Saccharomyces cerevisiae* Mcm2-7 subunits to alanine. Interestingly, only the PS1 hairpin of Mcm3 was essential for viability. While mutation of the PS1 hairpin in the remaining MCM subunits resulted in minimal phenotypes, with the exception of Mcm7 which showed slow growth under all conditions examined, the viable alleles were synthetic lethal with each other. Reconstituted Mcm2-7 containing Mcm3 with the PS1 mutation (Mcm3_K499A_) had severely decreased helicase activity. The lack of helicase activity provides a probable explanation for the inviability of the *mcm3*_K499A_ strain. The ATPase activity of Mcm2-7_3K499A_ was similar to the wild type complex, but its interaction with single-stranded DNA in an electrophoretic mobility shift assay and its associations in cells were subtly altered. Together, these findings indicate that the PS1 hairpins in the Mcm2-7 subunits have important and distinct functions, most evident by the essential nature of the Mcm3 PS1 hairpin in DNA unwinding.

## Introduction

In order for DNA replication to occur, the DNA duplex strands need to be separated by a replicative helicase [1]. Cellular replicative helicases tend to be hexameric rings that bind DNA within their central channels [2], [3]. The ring shape is thought to maintain association with DNA thus enhancing the processivity of the helicase [4], and may be important for DNA unwinding by potentially excluding one strand from the central channel [5], [6]. Regardless of the exact mechanism for DNA unwinding, the helicase must use nucleotide binding and hydrolysis to translocate along the bound DNA.

**Figure 1:**
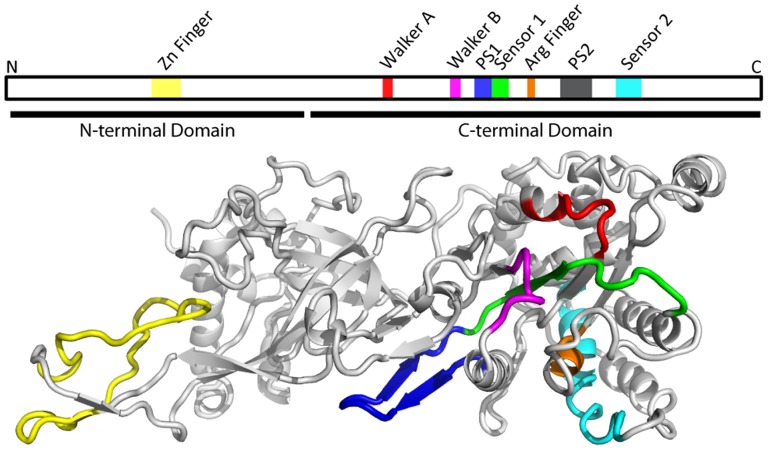
Structure of the Mcm proteins. Organization of the Mcm proteins in both the linear protein sequence (top) and in the folded proteins, based on the crystal structure of the *Solfolobus solfataricus* Mcm protein (PDB-ID 3F9V); [7]. The Mcm proteins are members of the AAA+ family of ATPases. The ATPase active sites are formed at the interface between two subunits. The Walker A (red), Walker B (magenta), and Sensor-1 (green) motifs are contributed by one subunit; the Arginine Finger (orange) and Sensor-2 (cyan) motifs are contributed by a second subunit (reviewed in 13). The Pre-Sensor 1 motif (PS1; blue) harbors a conserved lysyl residue at the turn between the two β-strands, and is not directly involved in ATP hydrolysis; this lysyl residue is the subject of the current work. For clarity, the PS2 motif is not indicated on the 3-dimensional structure.

X-ray structures of homo-hexameric replicative helicases that are members of the AAA+ family, including the superfamily 3 (SF3) helicase from bovine papillomavirus (E1) and mini-chromosome maintenance (MCM) from archaeal species, provide insight into how DNA translocation is achieved [7]–[10]. Notably, a β hairpin from each subunit projects into the central channel of the helicase. The structure of the E1 hexameric helicase with single-stranded DNA in its central channel identifies residues at the tip of the hairpin that contact the sugar phosphate backbone; in particular a lysine side-chain forms a salt-bridge with the DNA backbone [11]. ATP binding and hydrolysis are thought to drive conformational changes, leading to a sweeping motion of the β hairpins that moves DNA through the central channel [9]. Later structures of archaeal MCM proteins demonstrated the existence of the β hairpins with a lysine residue near the tip [7], [10]. These hairpins are referred to as the pre-sensor 1 (PS1) hairpins due to their position adjacent to the sensor 1 motif of the AAA+ domain as shown for the *Sulfolobus solfataricus* (Sso) MCM (Figure 1). Mutation of the conserved lysine in archaeal MCM proteins abrogates its helicase activity, but only slightly affects DNA binding, consistent with a role in DNA translocation [12].

In eukaryotic cells, the replicative helicase is comprised of six paralogous proteins of the AAA+ family, termed Mcm2-7. Each of the six subunits is essential for DNA replication in cells from yeast to mammals [13], [14]. The requirement for six distinct subunits may reflect the greater need for control of DNA replication and hence cell proliferation in eukaryotic cells compared to other systems. Indeed, the Mcm2-7 subunits are differentially targeted by protein kinases for control of cell proliferation [15]–[25], and have distinct roles in the activity of the intact complex [26]. In this regard, ATP sites found within each of the Mcm subunits are formed at the interface of neighboring subunits, and contribute differently to the overall ATPase activity of the complex [26]–[28]. Not all of the ATP sites are essential for DNA unwinding, even though the ATP sites are essential for viability [28]–[31]. Models for DNA unwinding by the homo-hexameric helicases suggest each subunit makes an identical contribution. This is not the case for Mcm2-7 as suggested by the distinct sequences of the components and the different ATPase activity of subunit pairs [26], [27]. However, the exact contribution each subunit makes to the DNA unwinding by Mcm2-7 is currently unknown.

Here, we have mutated the conserved lysine residue in the PS1 hairpin of each of the *Saccharomyces cerevisiae* Mcm2-7 subunits to alanine and examined the effect of the mutations. Interestingly, only the PS1 hairpin of Mcm3 is essential for viability. Mutation of the PS1 hairpin in Mcm7 resulted in growth related phenotypes, and strains with pairwise mutations in the remaining PS1 hairpins displayed synthetic slow or lethal interactions. Consistent with the observed loss of viability, Mcm2-7 complexes containing Mcm3 bearing the PS1 mutation (Mcm3_K499A_) show decreased DNA unwinding *in vitro*. The Mcm3_K499A_-containing Mcm2-7 has reduced binding to single-stranded DNA in an electrophoretic mobility shift assay, and analysis of Mcm3_K499A_ in yeast cell extracts revealed differences in its molecular associations. Together our results indicate the importance of the PS1 hairpins in Mcm2-7 function, and identify an essential function of Mcm3.

## Materials and Methods

### Plasmids

Oligonucleotides used to construct plasmids are listed in Table 1. For plasmid shuffling of the *MCM* genes, two plasmids for each wild-type gene were constructed (plasmids are listed in Table 2). The first set (pMD264, 245, 244, 227 238 and 228, representing *MCM2* through *7*, respectively) in the *URA3*-containing centromeric plasmid YCplac33 [32] were amplified by PCR using a UTR primer and coding primer, and contained the promoter region and coding sequence for each *MCM*. Oligonucleotide pairs MD81/MD82, MD83/MD84, MD85/MD86, MD87/MD88, MD89/MD90, and MD90/MD91 were used to amplify *MCM2, MCM3, MCM4, MCM5, MCM6*, and *MCM7*, respectively. The amplified *MCM* was then ligated into YCplac33 using the restriction enzyme cut sites indicated with the primers in Table 1. The second set of wild-type genes were cloned in YCplac111 (*LEU2-CEN*; [32]). For *MCM3* through *MCM6* these were constructed in a two-step process. The promoter region was amplified by PCR using a UTR primer and a start primer, then inserted into YCplac111 using the restriction sites in Table 1 (pMD229, 230, 232 and 240). A *Blp*I linker was then inserted into the polylinker *Sma*I site of the vector for *MCM4* and *MCM5* (pMD242 and 237). The remaining coding region was then inserted from a pET expression plasmids [27] using *Nde*I-*Sac*I for *MCM3* (pMD235), *Nde*I-*Blp*I for *MCM4* and *MCM5* (pMD379 and 378), and *Nde*I-*Bam*HI for *MCM6* (pMD239). For *MCM7*, the promoter region plus coding sequence up to the *Sal*I site at 251 base pairs was amplified by PCR using oligonucleotides (MD91 and 113) and cloned into YCplac111 (pMD241). pMD241 was digested with *Sma*I and a *Blp*I linker inserted to generate pMD260. The remaining coding sequence of *MCM7* was inserted from a pET expression plasmid [27] using *Sal*I and *Blp*I to give pMD261. To generate *mcm3_K499A_*- (pMD386; MD432/MD433), *mcm4_K658A_*- (pMD391; MD411/MD412), *mcm5_K506A_*- (pMD411; MD434/435), *mcm6_K665A_*- (pMD358; MD413/MD414), *mcm3_K499R_*- (MD501; MD612/MD613), *mcm3_K499Q_*- (pMD558; MD661/MD662), and *mcm3_K499N_*- (pMD559; MD663/MD664) YCplac111, the Stratagene QuikChange site-directed mutagenesis kit was used with the indicated primer pairs. Isolated clones were sequenced.

**Table 1:** Oligonucleotides used in this study.

#	Description	Site	Sequence (5′-3′)
*MD81*	*MCM2* UTR	*Sph*I	TTGGTCGCATGCACTTTTCATCTAAATGGATTA
*MD82*	*MCM2* coding	*Sac*I	TAGTGTGAGCTCTTATCCAGATATTCGTAGGAA
*MD83*	*MCM3* UTR	*Sph*I	AAGGTCGCATGCGTTATTTTTCTCTTTTTTTTCAA
*MD84*	*MCM3* coding	*Sac*I	TAGTGTGAGCTCAGTAAACATTCCTGTGACAT
*MD85*	*MCM4* UTR	*Pst*I	TTAGCTCTGCAGACTTGAACGGATCTTTAGTAT
*MD86*	*MCM4* coding	*Sac*I	TAGTGTGAGCTCGGAATGATTGTAGTAGACAG
*MD87*	*MCM5* UTR	*Sph*I	TTGGTCGCATGCTTTGTAAAAACAAAGAGTAAAATT
*MD88*	*MCM5* coding	*Sma*I	TATTATCCCGGGAAGGCGTCAAGCTAAGAC
*MD89*	*MCM6* UTR	*Pst*I	TTAGCTCTGCAGTTGAAAAAAACCAGTTTTAACC
*MD90*	*MCM6* coding	*Bam*HI	TATTATGGATCCATCCGCAAGAGTGCACTG
*MD91*	*MCM7* UTR	*Sph*I	TTGGTCGCATGCAAGGAAAGGCCGTTTTT
*MD92*	*MCM7* coding	*Sma*I	TATTATCCCGGGAAAGAATGAAGGCCCTGT
*MD109*	*MCM3* start	*Sal*I	TTAGTCGTCGACATATGTAATTGACGTTTGTATCTTTT
*MD110*	*MCM4* start	*Sac*I	TTAGTCGAGCTCATATGTTTTAAGTTCTTGAGGTTC
*MD111*	*MCM5* start	*Sma*I	TTAGTCCCGGGCATATGTTATCTGGCTTCTAATTCAC
*MD134*	*MCM6* start	*Xba*I	ATAATCTAGACATATGAAAAAAACCAGTTTTAACCT
*MD113*	*MCM7*	*Sal*I	ATTATAGTCGACAGGAAGC
*MD274*	*MCM4* coding	*Bgl*II	TGATTGTAGAGATCTTCAGACACGGTTATTCAG
*MD411*	*MCM4_K658A_* coding	*Msp*A1I	GCAGACTATTTCAATCGCAGCAGCGGGAATTATCACAACAC
*MD412*	*MCM4_K658A_* noncoding	*Msp*A1I	GTGTTGTGATAATTCCCGCTGCTGCGATTGAAATAGTCTGC
*MD413*	*MCM6_K665A_* coding	*Pst*I	CAGACCATCTCTATTGCTGCAGCTGGTATTCACGCTAC
*MD414*	*MCM6_K665A_* noncoding	*Pst*I	GTAGCGTGAATACCAGCTgcAGCAATAGAGATGGTCTG
*MD434*	*MCM5_K506A_* coding	*Alw*NI	ACAATCTCCATCGCAGCAGCTGGTATCACTACAGTGC
*MD435*	*MCM5_K506A_* noncoding	*Alw*NI	GCACTGTAGTGATACCAGCTGCTGCGATGGAGATTGT
*MD432*	*MCM3_K499A_* coding	*Sac*II	CAAACGGTGACGATTGCCGCGGCAGGTATTCACACAAC
*MD433*	*MCM3_K499A_* noncoding	*Sac*II	GTTGTGTGAATACCTGCCGCGGCAATCGTCACCGTTTG
*MD556*	*MCM3* coding	*Not*I	ATGACGCGGCCGCCATGGAAGGCTCAACGGGATT
*MD612*	*MCM3_K499R_* coding		AAACGGTGACGATTGCCCGGGCAGGTATTCACACAACA
*MD613*	*MCM3_K499R_* noncoding		TGTTGTGTGAATACCTGCCCGGGCAATCGTCACCGTTT
*MD661*	*MCM3_K499Q_* coding		ACAAACGGTGACGATTGCCCAAGCAGGTATTCACACAA
*MD662*	*MCM3_K499Q_* noncoding		TTGTGTGAATACCTGCTTGGGCAATCGTCACCG TTTGT
*MD663*	*MCM3_K499N_* coding		AAACGGTGACGATTGCCAATGCAGGTATTCACACAACA
*MD664*	*MCM3_K499N_* noncoding		TGTTGTGTGAATACCTGCATTGGCAATCGTCACCGTTT
*MD659*	*Flag^3^* coding	*Nde*I *Not*I overhangs	TATGGATTATAAAGATGATGATGATAAAGCTGCTGATTATAAAGATGATGATGATAAAGCTGCTGATTATAAAGATGATGATGATAAAGC
*MD660*	*Flag^3^* noncoding	*Nde*I *Not*Ioverhangs	GGCCGCTTTATCATCATCATCTTTATAATCAGCAGCTTTATCATCATCATCTTTATAATCAGCAGCTTTATCATCATCATCTTTATAATCCA

**Table 2:** Plasmids used in this study.

Plasmid	Description
pMD264 [30]	*MCM2-*YCplac33
pMD245	*MCM3*-YCplac33
pMD244	*MCM4*-YCplac33
pMD227	*MCM5-*YCplac33
pMD238	*MCM6-*YCplac33
pMD228	*MCM7-*YCplac33
pMD229	*MCM3* 5′UTR-YCplac111
pMD230	*MCM4* 5′UTR-YCplac111
pMD232	*MCM5* 5′UTR-YCplac111
pMD240	*MCM6* 5′UTR-YCplac111
pMD241	*MCM7* 5′UTR-YCplac111
pMD266	*MCM2-*YCplac33-pLU9
pMD235	*MCM3*-YCplac111
pMD242	*MCM4* 5′UTR-YCplac111 (*Blp*I)
pMD379	*MCM4* YCplac111
pMD237	*MCM5* 5′UTR-YCplac111 (*Blp*I)
pMD378	*MCM5*-YCplac111
pMD239	*MCM6-*YCplac111
pMD260	*MCM7* 5′UTR-YCplac111 (*Blp*I)
pMD261	*MCM7*-YCplac111
pMD307	*mcm2_K633A_*-YCplac111
pMD386	*mcm3_K499A_*-YCplac111
pMD391	*mcm4_K658A_*-YCplac111
pMD411	*mcm5_K506A_*-YCplac111
pMD358	*mcm6_K665A_*-YCplac111
pMD308	*mcm7_K550A_*-YCplac111
pMD438	*mcm4_K658A_*-YIplac211
pMD439	*mcm5_K506A_*-YIplac211
pMD440	*mcm6_K665A_*-YIplac211
pMD346	*mcm7_K550A_*-YIplac211
pMD466	*mcm3_K499A_*-pET24a
pMD501	*mcm3_K499R_*-YCplac111
pMD558	*mcm3_K499Q_*-YCplac111
pMD559	*mcm3_K499N_*-YCplac111
pMD502	*DED1-myc^9^-MCM3*-YCplac111
pMD503	*DED1-myc^9^-mcm3_K499A_*-YCplac111
pMD407	*GAL10-*YCplac111
pMD554	*GAL10-Flag^3^*-*mcm3_K499A_*-YCplac111
pMD560	*GAL10-Flag^3^*-*MCM3*-YCplac111
pMD561	*mcm3_K499A_*-YIplac211
pMD562	*MCM3*-YEplac181
pMD563	*mcm3_K499A_*-YEplac181

To clone *MCM3* (pMD562) and *mcm3_K499A_* (pMD563) into YEplac181, a *Sph*I-*Sac*I fragment of pMD235 and pMD386 was ligated into the same sites of YEplac181. A fragment of *Sph*I-*Sac*I from pMD386 was also ligated to the same sites in YIplac211 to generate *mcm3_K499A_*-YIplac211 (pMD561). For cloning *mcm4_K658A_* into YIplac211, we first amplified *mcm4_K658A_* by PCR using oligonucleotides MD85, MD274, and *mcm4_K658A_*-YCplac111 as template. This product was cloned into YIplac211 using *Pst*I and *Bam*HI to give pMD438. For *mcm5_K506A_*, a *Blp*I site was introduced into YIplac211 at the *Sma*I site. *mcm5_K506A_* was introduced into this plasmid from *mcm5_K506A_*-YCplac111 as a *Sph*I-*Blp*I fragment (pMD439). pMD440 was constructed by inserting a *Sph*I-*Bam*HI fragment of *mcm6_K665A_*-YCplac111 into YIplac211. To generate *MCM3* and *mcm3_K499A_* myc^9^ N-terminally tagged expression plasmids, *MCM3* and *mcm3_K499A_* were amplified by PCR using oligonucleotides MD84, MD556, and cloned using *Not*I and *Sac*I into a derivative of YCplac111 where the *DED1* promoter drives expression of a myc^9^ N-terminally tagged protein [33]. The pET24a-*mcm3_K499A_* was cloned by cutting *mcm3_K499A_*-YCplac111 with *Nde*I and *Sac*I and ligating into the same sites of pET24a.

To construct *MCM3 and mcm_3K499A_* Flag^3^-tagged expression plasmids, a *GAL10* promoter containing YCplac111 plasmid (pMD407) was linearized with *Nde*I and *Sac*I. Oligonucleotides MD659/MD660 were annealed and ligated into linearized pMD407. *MCM3* and *mcm3*_K499A_ isolated from pMD502 and pMD503 were then inserted as *Not*I-*Sac*I fragments to give pMD560 and pMD554.

### Plasmid shuffling

Diploid heterozygous strains containing a KanMX deletion of a *mcm* gene were obtained from Open Biosystems. The *mcm2::his3* disruption strain (MDY54) was a derivative of YMD33 [31]. Each of these was transformed with the relevant *MCM*-YCplac33 plasmid and sporulated to give MDY16, 17, 40, 41, 70, and 100. *mcm* deletion haploid strains containing their corresponding *MCM*-YCplac33 were transformed with a *mcm_KA_*-YCplac111 or *MCM*-YCplac111. The transformed strains were grown in YPD, then plated on 5-FOA-containing media to select for cells that lost the *MCM*-YCplac33 [34].

### Yeast strains

All yeast strains are listed in Table 3. Two-step gene replacement was used to integrate PS1 hairpin mutation into the yeast genome [35]: *mcm2_K633A_* (MDY225 and 226), *MCM3*/*mcm3_K499A_* (MDY411), *mcm4*_K658A_ (MDY220), *mcm5_K506A_* (MDY221 and 222), *mcm6_K665A_* (MDY258 and 259), *mcm7_K550A_* (MDY253 and 254). Each mutation incorporated a unique restriction site (Table 1) for identification. YIplac211 plasmids were linearized with *Msc*I (*mcm3*), *Age*I, (*mcm4*) *Bsp*EI (*mcm5*), *Msc*I (*mcm6*), or *Bam*HI (*mcm7*) and transformed into BY4743. *URA3* positive colonies were grown in YPD liquid media, and then selected on 5-FOA-containing media. PCR amplification of the *MCM* loci was performed and restriction mapping used to confirm integration of *mcm_KA_*. The heterozygous diploid strains were sporulated and haploid *mcm_KA_* mutants isolated.

**Table 3:** Yeast strains used in this study.

Yeast strain	Genotype
MDY16	*MAT* **a** *his3Δ1 leu2Δ0 met15Δ0 ura3Δ0 mcm3Δ::KanMX* (YCplac33 *MCM3 URA3*)
MDY17	*MAT* **a** *his3Δ1 leu2Δ0 met15Δ0 ura3Δ0 mcm4Δ::KanMX* (YCplac33 *MCM4 URA3*)
MDY40	*MAT* **a** *his3Δ1 leu2Δ0 met15Δ0 ura3Δ0 mcm7Δ::KanMX* (YCplac33 *MCM7 URA3*)
MDY41	*MAT* **a** *his3Δ1 leu2Δ0 met15Δ0 ura3Δ0 mcm6Δ::KanMX* (YCplac33 *MCM6 URA3*)
MDY54	*MAT* **α** *leu2Δ0 MET15 ura2Δ0* lys2*Δ0 mcm2*::*his3* (YCplac33 *MCM2 URA3*)
MDY70	*MAT* **a** *his3Δ1 leu2Δ0 met15Δ0 ura3Δ0 mcm2Δ::his3* (YCplac111 *MCM2 LEU2*)
MDY71	*MAT* **a** *his3Δ1 leu2Δ0 met15Δ0 ura3Δ0 mcm2Δ::his3* (YCplac111 *mcm2_K633A_ LEU2*)
MDY72	*MAT* **a** *his3Δ1 leu2Δ0 met15Δ0 ura3Δ0 mcm7Δ::KanMX* (YCplac111 *MCM7 LEU2*)
MDY73	*MAT* **a** *his3Δ1 leu2Δ0 met15Δ0 ura3Δ0 mcm7Δ::KanMX* (YCplac111 *mcm7_K550A_ LEU2*)
MDY100	*MAT* **a** *his3Δ1 leu2Δ0 met15Δ0 ura3Δ0 mcm5Δ::KanMX* (YCplac33 *MCM5 URA3*)
MDY153	*MAT* **a** *his3Δ1 leu2Δ0 met15Δ0 ura3Δ0 mcm3Δ::KanMX* (YCplac111 *MCM3 LEU2*)
MDY154	*MAT* **a** *his3Δ1 leu2Δ0 met15Δ0 ura3Δ0 mcm4Δ::KanMX* (YCplac111 *MCM4 LEU2*)
MDY155	*MAT* **a** *his3Δ1 leu2Δ0 met15Δ0 ura3Δ0 mcm4Δ::KanMX* (YCplac111 *mcm4_K658A_ LEU2*)
MDY172	*MAT* **a** *his3Δ1 leu2Δ0 met15Δ0 ura3Δ0 mcm5Δ::KanMX* (YCplac111 *MCM5 LEU2*)
MDY173	*MAT* **a** *his3Δ1 leu2Δ0 met15Δ0 ura3Δ0 mcm5Δ::KanMX* (YCplac111 *mcm5_K506A_ LEU2*)
MDY220	*MAT* **a** *his3Δ1 leu2Δ0 ura3Δ0 mcm4_K658A_*
MDY221	*MAT* **α** *his3Δ1 leu2Δ0 ura3Δ0 mcm5_K506A_*
MDY222	*MAT* **a** *his3Δ1 leu2Δ0 ura3Δ0 mcm5_K506A_*
MDY225	*MAT* **α** *his3Δ1 leu2Δ0 ura3Δ0 mcm2_K633A_*
MDY228	*MAT* **α** *his3Δ1 leu2Δ0 ura3Δ0 mcm4_K658A_ mcm5_K506A_*
MDY229	*MAT* **a** *his3Δ1 leu2Δ0 ura3Δ0 mcm4_K658A_ mcm5_K506A_*
MDY253	*MAT* **α** *his3Δ1 leu2Δ0 ura3Δ0 mcm7_K550A_*
MDY254	*MAT* **a** *his3Δ1 leu2Δ0 ura3Δ0 mcm7_K550A_*
MDY258	*MAT* **α** *his3Δ1 leu2Δ0 ura3Δ0 mcm6_K665A_*
MDY259	*MAT* **a** *his3Δ1 leu2Δ0 ura3Δ0 mcm6_K665A_*
MDY402	*MAT* **a** *his3Δ1 leu2Δ0 met15Δ0 ura3Δ0 mcm3Δ::KanMX* (YCplac111 *mcm3_K499R_ LEU2*)
MDY403	*MAT* **a** *his3Δ1 leu2Δ0 met15Δ0 ura3Δ0 mcm6Δ::KanMX* (YCplac111 *MCM6 LEU2*)
MDY404	*MAT* **a** *his3Δ1 leu2Δ0 met15Δ0 ura3Δ0 mcm6Δ::KanMX* (YCplac111 *mcm6_K663A_ LEU2*)
MDY405	*MAT* **a** *his3Δ1 leu2Δ0 met15Δ0 ura3Δ* (YCplac111 *DED1-myc^9^-MCM3*)
MDY406	*MAT* **a** *his3Δ1 leu2Δ0 met15Δ0 ura3Δ* (YCplac111 *DED1-myc^9^-mcm3_K499A_*)
MDY407	*MAT* **a** *his3Δ1 leu2Δ0 met15Δ0 ura3Δ* (YCplac111 *GAL10*-*Flag^3^*-*MCM3 LEU2*)
MDY408	*MAT* **a** *his3Δ1 leu2Δ0 met15Δ0 ura3Δ* (YCplac111 *GAL10*-*Flag^3^*-*mcm3_K499A_ LEU2*)
MDY411	*MAT* **a**/**α** *his3Δ1/his3Δ1 leu2Δ0/leu2Δ0 lys2Δ0/LYS2 met15Δ0/MET15 ura3Δ0/ura3Δ0 MCM3*/*mcm3_K499A_*
MDY414	*MAT* **a** *his3Δ1 leu2Δ0 met15Δ0 ura3Δ0 mcm3Δ::KanMX* (YEplac181 *MCM3 LEU2*)
BY4741 [52]	*MAT* **a** *his3Δ1 leu2Δ0 met15Δ0 ura3Δ*
BY4743 [52]	*MAT* **a**/**α** *his3Δ1/his3Δ1 leu2Δ0/leu2Δ0 lys2Δ0 met15Δ0 ura3Δ0/ura3Δ0*

### Imaging yeast overexpressing of *MCM3* and *mcm3_K499A_*

BY4741 transformed with YCplac111-*GAL10-MCM3* or YCplac111-*GAL10-mcm3_K499A_* was grown in minimal media lacking leucine supplemented with 2% galactose overnight. The overnight cultures were diluted to 10^6^ cells/mL with minimal media lacking leucine supplemented with 2% galactose. After two hours cells were imaged under bright field using a Nikon Eclipse Ti microscope. Measurements were taken using NIS Elements Imaging Software.

### Proteins

The recombinant Mcm subunits were purified from *Escherichia coli* and reconstituted into Mcm2-7 as described [27].

### Mcm3_K499A_ purification

The *mcm3_K499A_* pET24a plasmid was transformed into BL21 DE3 Codon+. Twelve liters of transformed cells were grown in LB media with 100 mg/L of ampicillin, and 25 mg/L of chloramphenicol to a density of A_600_ = 0.6. Cells were cooled to 15°C and isopropyl β-D-1-thiogalactopyranoside added to a final concentration of 1 mM. Cells were incubated at 15°C for 20 hours prior to collecting the cell pellet. The cell pellet was resuspended in 250 mL of Buffer H (20 mM HEPES, pH 7.5, 2 mM DTT, 10% v/v glycerol, and 0.1 mM EDTA) and lysed at 15000 psi in an Emulsiflex-C3 high pressure homogenizer. Debris was pelleted by centrifugation at 15000 g for 25 minutes and the supernatant decanted. Ammonium sulfate was added to the supernatant (0.25 g/mL) with stirring at 4°C. Ammonium sulfate precipitate was collected by centrifugation, and resuspended with 150 mL of Buffer H. The solution was dialyzed overnight at 4°C in 4 L of Buffer H with stirring and then loaded onto a Fast flow Q Sepharose column equilibrated with Buffer H and washed with 7 column volumes of Buffer H containing 50 mM NaCl. Mcm3_K499A_ was eluted over 10 column volumes in a gradient of 0–500 mM NaCl in Buffer H. Fractions containing Mcm3_K499A_ were collected and dialyzed overnight in 750 mL of Buffer H at 4°C. A single-stranded (ss) DNA-Sepharose column was made by coupling boiled and sonicated salmon sperm DNA to cyanogen bromide-activated Sepharose 4B (GE Life Sciences). The dialyzed solution was loaded onto the ssDNA-Sepharose column equilibrated with Buffer H, washed with 10 column volumes and protein eluted with 10 column volumes of a 0–500 mM NaCl gradient in Buffer H. The fractions containing Mcm3_K499A_ were pooled and ammonium sulfate added (0.3 g/mL) with stirring at 4°C. The precipitate was collected by centrifugation, resuspended and dialyzed against 400 mL of Buffer A (20 mM Tris-HCl, pH 7.5, 2 mM DTT, 10% v/v glycerol, and 0.1 mM EDTA). The solution was loaded onto a MonoQ (GE Life Sciences) column equilibrated with Buffer A containing 50 mM NaCl, and the protein eluted over 20 column volumes of a 0–500 mM NaCl gradient in Buffer A. Fractions containing Mcm3_K499A_ were pooled and dialyzed in one liter of Buffer H with stirring, overnight at 4°C. The dialyzed solution was loaded onto a MonoS (GE Life Sciences) column equilibrated with Buffer H containing 50 mM NaCl, and the protein eluted over 20 column volumes of a 0–500 mM NaCl gradient in Buffer H. Fractions were collected and frozen at −80°C.

### Western blotting

Western blotting was performed using polyvinylidene difluoride membranes and anti-myc (Sigma-Aldrich) as described by Mutiu *et al* [36].

### Biochemical assays

DNA unwinding and ATPase assays were performed essentially as described by Stead *et al* [25] with the exception that intact complex was used. ATP hydrolysis was assayed using thin-layer chromatography. Each 15-µl reaction contained 1 mM [γ^32^P]ATP (20 mCi/mmole; Perkin Elmer Life Sciences), 20 mM Tris–HCl (pH 7.5), 10 mM magnesium acetate, and 2 mM DTT, and 200 nM Mcm2-7. At the indicated times, 2 µL of each reaction was removed and quenched with 2 µL of 50 mM EDTA (pH 8). One microliter was spotted onto a polyethyleneimine cellulose sheet (EM Science), developed in 0.6 M potassium phosphate (pH 3.4), dried, exposed to a PhosphorStorage screen, and scanned with a Storm 860 scanner (GE Healthcare). DNA unwinding measurements were performed with a DNA substrate containing 30 nucleotides of duplex, with 60 nucleotides of single-stranded DNA on one strand and a 5′ biotin on the other strand. Each reaction (6 µL) contained 20 mM Tris–HCl (pH 7.5), 10 mM magnesium acetate, 100 µM EDTA, 5 mM DTT, 5 mM ATP, 67 nM streptavidin, 1 nM substrate with 100 nM, 200 nM or 400 nM Mcm2-7. Samples were analyzed by native PAGE using an 8% gel in Tris–borate–EDTA buffer.

### DNA binding assay

The single-stranded DNA affinity chromatography was performed with a 200 µL single-stranded DNA Sepharose column (see above Mcm3_K499A_ purification). Five micrograms of Mcm2-7 complex were applied to the column in buffer H containing 5 mM ATP and 50 mM NaCl and eluted with buffer containing 5 mM ATP and either 50 mM, 100 mM, 200 mM, 300 mM, 400 mM, or 500 mM NaCl. Each elution was performed twice with one column volume. 24 µL of each fraction was separated by SDS-PAGE (6%). The polyacrylamide gels were stained with colloidal blue stain, and then washed with deionized water to destain the gels for imaging. The destained gels were then silver stained according to protocol provided in Pierce Silver Stain Kit (Thermo Scientific) to detect protein in the column fractions.

The electrophoretic mobility shift assay was adapted from Stead *et al.* [25]. Briefly, Mcm2-7 and Mcm2-7_3K499A_ complexes were incubated with 1 nM of 5′-end ^32^P-labeled oligonucleotide (ATGTCCTAGCAAGCCAGAATTCGGCAGCGTC-(T)_60_) at 37°C in buffer containing 20 mM Tris–HCl (pH 7.5), 10 mM magnesium acetate, 100 µM EDTA, 5 mM DTT, and 5 mM ATP for 10 minutes. One microgram of anti-Mcm7 antibody (Santa Cruz Biotech) was added to another set of Mcm2-7 samples prior to incubation with radiolabeled oligonucleotide to disrupt Mcm2-7 binding. Four microliters of 12.5% glycerol was added to each reaction and then resolved in a 5% native (Tris–borate–EDTA) polyacrylamide gel (19∶1 acrylamide:bis–acrylamide; BioShop Canada) containing 5% glycerol, 0.1% NP-40 and 10 mM Mg(CH_3_COO)_2_ at 30 mA for 2.5 hours. The gel was dried, and exposed to film.

### Gel filtration chromatography

Proteins extracts were prepared cryogenically as described by Saleh *et al* [37]. Five mg of yeast extract prepared in 50 mM sodium phosphate pH 7.0, 150 mM NaCl was loaded at a flow rate of 0.3 mL/min. onto a 24 mL FPLC Superose 6HR10/30 column (Amersham Pharmacia Biotech.). Protein from 10 µL aliquots of 250 µL fractions for wild type protein and 10 µL for Mcm3_K449A_ were resolved by SDS-PAGE and proteins detected by western blotting.

### Modeling of *S. cerevisiae* Mcm2-7

Individual Mcm2 through Mcm7 subunits were modeled based on the 4.35-Å resolution structure of *Solfolobus solfataricus* Mcm (SsoMcm; PDB ID 3F9V; [7]). This was done using a multiple sequence alignment incorporating SsoMcm residues 9 to 603 and residues 204 to 849 of Mcm2; 22 to 744 of Mcm3; 188 to 837 of Mcm4; 25 to 692 of Mcm5; 109 to 839 of Mcm6; and 15 to 728 of Mcm7. The comparative modeling protocol of Rosetta was used to thread the sequences onto the SsoMcm structure, build loop regions and additional domains in the Mcm2 through Mcm7 subunits that were not present in SsoMcm, and refine the overall structure of the subunits [38],[39]. A model for the Mcm2-7 hexamer was then assembled by superimposing the N-terminal domains of the Mcm2 through Mcm7 subunits on the N-terminal domains of the *Methanobacterium thermoautotrophicum* hexamer (PDB ID 1LTL; [40]). Molecular graphics were generated using PyMOL (Version 1.5.0.5, Schrödinger, LLC) and electrostatic surface calculations were carried out using PDB2PQR [41] and APBS [42].

## Results and Discussion

### Effects of PS1 mutations in Mcm2-7 on yeast growth

**Figure 2:**
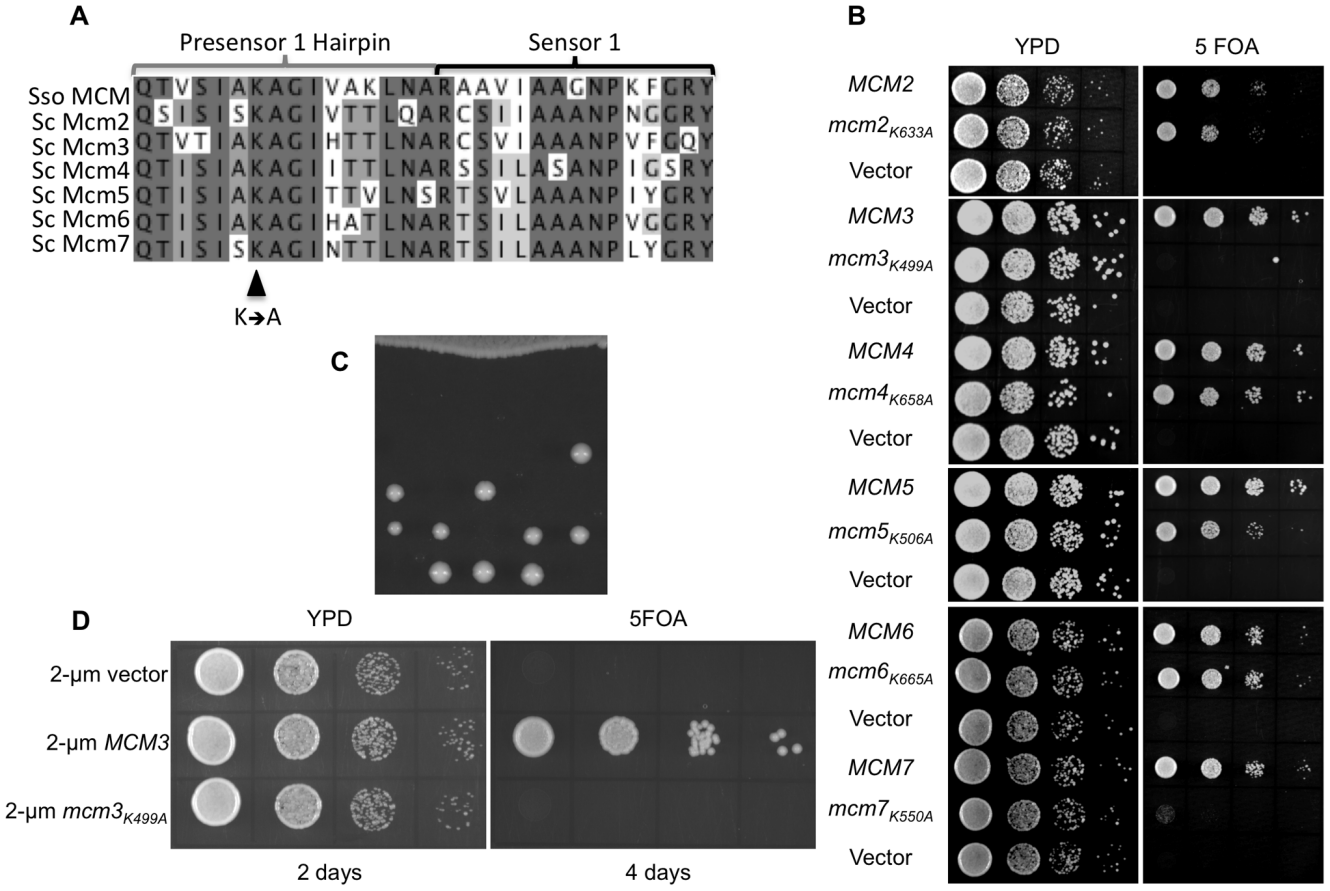
Growth of strains bearing the PS1 hairpin alleles. (A) Alignment of the PS1 hairpin and Sensor 1 in SsoMcm with *S. cerevisiae* Mcm2-7 using T-Coffee. (B) Plasmid shuffling of Mcm PS1 hairpin mutations. The wild-type Mcm gene (*MCM*), the Mcm gene with PS1 hairpin mutation (*mcm_KA_*), or the empty *LEU2-CEN* plasmid (Vector) were transformed into a haploid yeast strain deleted for the genomic copy of the corresponding Mcm gene and containing a copy of the gene on a *URA3-CEN* plasmid. Transformed yeast were grown overnight at 30°C in YPD media, serially diluted, and then spotted onto a YPD plate or a plate containing 5-FOA. (C) The diploid strain MDY411 (*MCM3*/*mcm3_K499A_*) was sporulated and tetrads dissected. The dissection plates (YPD) were incubated at 30°C. (D) MDY16 (*mcm3Δ::KanMX* YCplac33 *MCM3 URA3*) was transformed with 2 micron plasmid YEplac181 (2-µm Vector), pMD562 (2-µm *MCM3*), or pMD563 (2-µm *mcm3_K499A_*). Transformants were grown overnight at 30°C in YPD media, serially diluted, and then spotted onto a YPD plate or a plate containing 5-FOA.

Each of the Mcm subunits contains a pre-sensor 1 (PS1) hairpin adjacent to the sensor 1 motif of the AAA+ domain (Figure 1). To determine whether the PS1 hairpin motifs are important for the function of Mcm2-7, the replicative helicase in eukaryotic cells, we mutated the conserved lysine residue to alanine in each of the Mcm subunits (Figure 2A). The mutant genes, encoded on *LEU2-*containing centromeric plasmids, were shuffled into haploid strains bearing a deletion in the corresponding *mcm* and maintained by *MCM* on a *URA3-*containing centromeric plasmid. Lack of function of the mutant Mcm subunit is indicated by the absence of growth on media containing 5-fluoroorotic acid (5-FOA), which is toxic to *URA3*-expressing strains. Of the six subunits, only a mutation in the Mcm3 PS1 hairpin (*mcm3_K499A_*) resulted in a loss of viability on 5-FOA (Figure 2B). Slow growth was noted for the *mcm7_K550A_* strain, but it is viable. To more fully compare the relative phenotypes of the *mcm3_K499A_* and *mcm7_K550A_* strains, we have also incubated the strains for an extended period (Figure S1). The presence of the mutant allele as the sole copy of the *mcm_KA_* in the viable strains was confirmed by PCR and restriction digestion.

The Mcm genes were first identified through their requirement for the maintenance of autonomously replicating chromosomes in yeast [43]. To ensure that the inviability of the *mcm3_K499A_* strain was not due to a failure to maintain the plasmid, we integrated the *mcm3_K499A_* mutation into the diploid yeast strain BY4743 and analyzed the viability of spore colonies after sporulation and tetrad dissection. As shown in Figure 2C viability segregates in a 2∶2 manner consistent with the inability of *mcm3_K499A_* to support growth. We also addressed whether *mcm3_K499A_* would support growth when overexpressed on a 2-micron plasmid by plasmid shuffling (Figure 2D). Similar to what we observed with the centromeric plasmid, no growth was detected. Further suggesting that the inability of *mcm3_K499A_* to support viability is not the result of reduced stability of the protein, we find that myc^9^-tagged wild-type Mcm3 and Mcm3_K499A_ are found at a similar level (Figure S2). Taken together we conclude that the Mcm3 PS1 hairpin is essential for the function of the Mcm2-7 complex.

**Figure 3:**
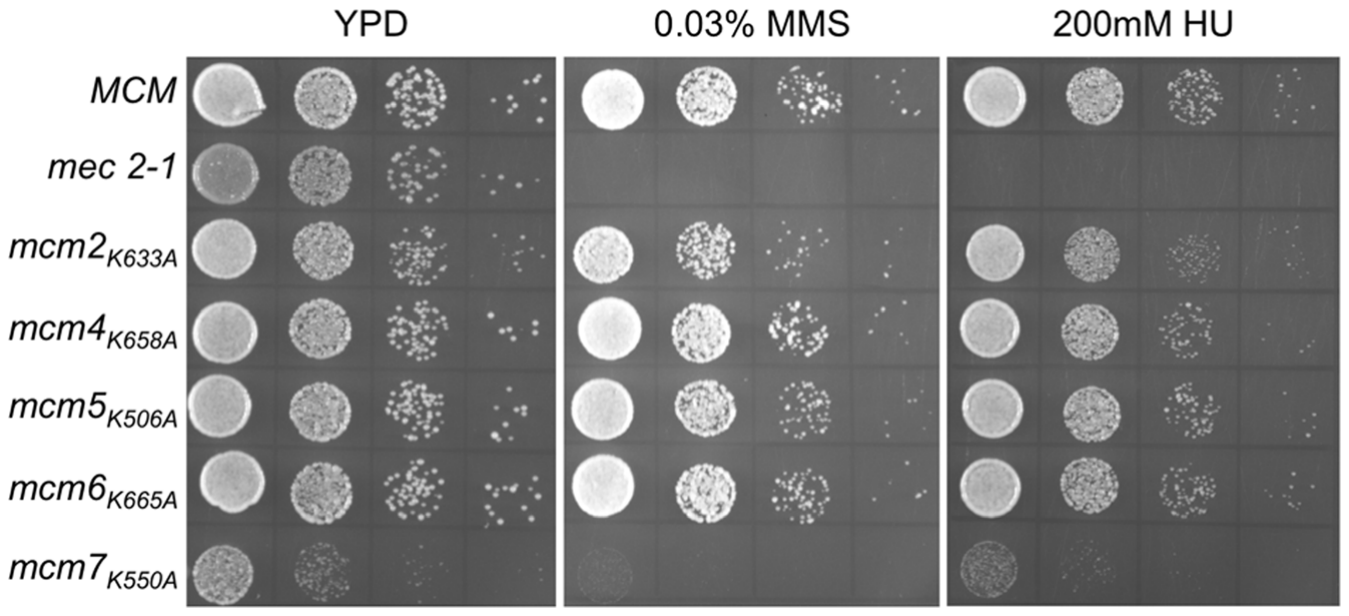
Effect of temperature and genotoxic agents on PS1 hairpin mutants. (A) Cultures of yeast strains BY4741 (wild-type), MDY225 (*mcm2_K633A_*), MDY220 (*mcm4_K658A_*), MDY222 (*mcm5_K506A_*), MDY256 (*mcm6_K665A_*), and MDY254 (*mcm7_K550A_*) were grown overnight in YPD at 30°C, serially diluted 10-fold, spotted onto YPD plates and incubated at either 16°C, 30°C or 37°C. (B) Cultures of yeast strains BY4741 (wild-type), MDY225 (*mcm2_K633A_*), MDY220 (*mcm4_K658A_*), MDY222 (*mcm5_K506A_*), MDY256 (*mcm6_K665A_*), and MDY254 (*mcm7_K550A_*) were grown in YPD, and 10-fold serial dilutions spotted onto YPD and YPD containing either 0.03% methyl methanesulfonate (MMS) or 200 mM hydroxyurea (HU). Plates were incubated at 30°C. A *mec2-1* [51] strain, known to be sensitive to genotoxic stress, was also spotted on the plates.

To further characterize the effects of the PS1 mutations, we examined the growth of viable strains bearing the mutations at different temperatures (Figure 3A). For these experiments the PS1 hairpin mutations were integrated into the genome. At each of the temperatures, the relative growth *mcm7_K550A_* was reduced. The *mcm2_K633A_* containing strain grew somewhat more slowly at 16°C. Mutations in the Mcm subunits often result in sensitivity to genotoxic agents. Therefore, we examined the growth of each of the strains with a PS1 mutation on media containing the ribonucleotide reductase inhibitor hydroxyurea, or the DNA-damaging agent methyl methanesulfonate (MMS) (Figure 3B). Consistent with its slow growth at different temperatures, strains containing *mcm7_K550A_* grew slowly on both agents, with a slight sensitivity to MMS noted. Similarly strains bearing the other PS1 mutations were not sensitive to hydroxyurea or MMS.

**Figure 4:**
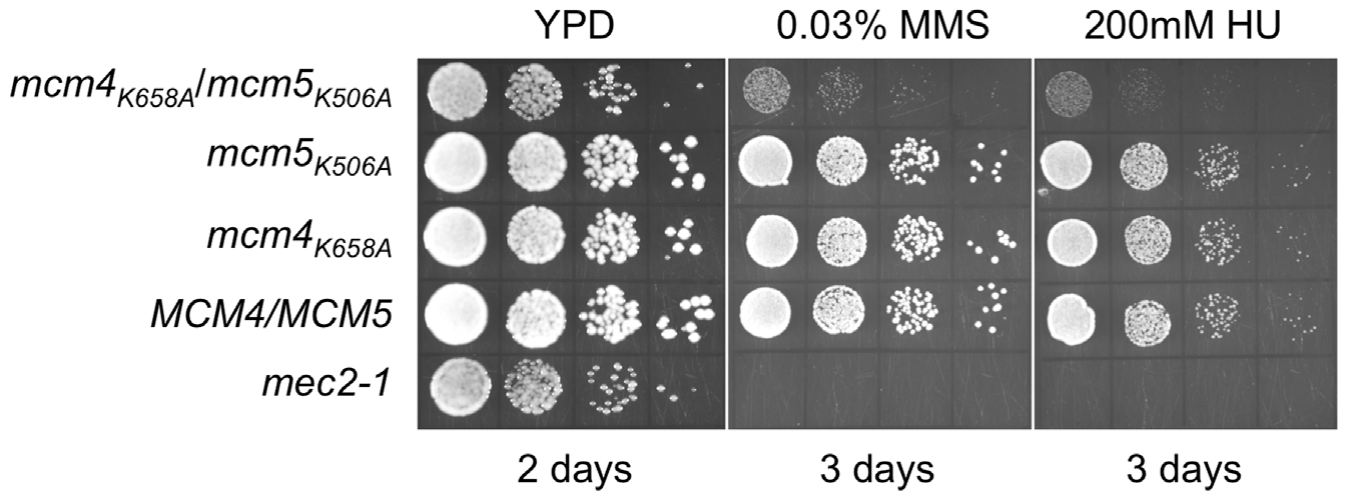
Phenotype of the *mcm4_K658A_*/*mcm5_K506A_* double mutant strain. Haploid yeast strains containing either PS1 mutations in both Mcm4 and Mcm5 (MDY229), single PS1 mutations in either Mcm4 (MDY220) or Mcm5 (MDY222), and the wild-type strain BY4741 were grown overnight at 30°C in liquid YPD, serially diluted 10-fold, and then spotted onto YPD, YPD containing 0.03% methyl methanesulfonate (MMS), or 200 mM hydroxyurea (HU). A *mec2-1* [51] strain, known to be sensitive to genotoxic stress, was also spotted on the plates.

Our plasmid shuffling experiments indicate that a single mutation of the conserved PS1 lysine (K499) residue in Mcm3 results in loss of viability. In contrast, the homo-hexameric *S. solfataricus* Mcm (SsoMcm) accommodates several subunits with disruptions in catalytic elements and still maintains significant helicase activity [44]. Therefore, we investigated the effect of mutating two different PS1 hairpins in the Mcm2-7 complex. We mated the haploid strains containing individual PS1 hairpin mutations to produce all the possible pair-wise combinations. After sporulating the heterozygous strains, we screened the spore colonies for viable double mutants. Only spore colonies with *mcm4_K658A_* and *mcm5_K506A_* were viable (Table 4). These grew more slowly than wild-type or strains containing either *mcm4_K658A_* or *mcm5_K506A_* and were more sensitive to hydroxyurea and MMS (Figure 4).

**Table 4:** Synthetic lethal crosses of *mcm* PS1 alleles.

Cross	Spore colonies examined^1^	Viable with two mutations	P - value
*mcm2_K633A_* x *mcm4_K658A_*	24	0	0.002
*mcm2_K633A_* x *mcm5_K506A_*	21	0	0.007
*mcm2_K633A_* x *mcm6_K665A_*	18	0	0.007
*mcm2_K633A_* x *mcm7_K550A_*	19	0	0.007
*mcm4_K658A_* x *mcm5_K506A_*	24	6	0.161
*mcm4_K658A_* x *mcm6_K665A_*	16	0	0.018
*mcm4_K658A_* x *mcm7_K550A_*	15	0	0.018
*mcm5_K506A_* x *mcm6_K665A_*	19	0	0.007
*mcm5_K506A_* x *mcm7_K550A_*	21	0	0.007
*mcm6_K665A_* x *mcm7_K550A_*	24	0	0.002

^1^ A random spore analysis was performed by isolating individual spore colonies from tetrads.

**Figure 5:**
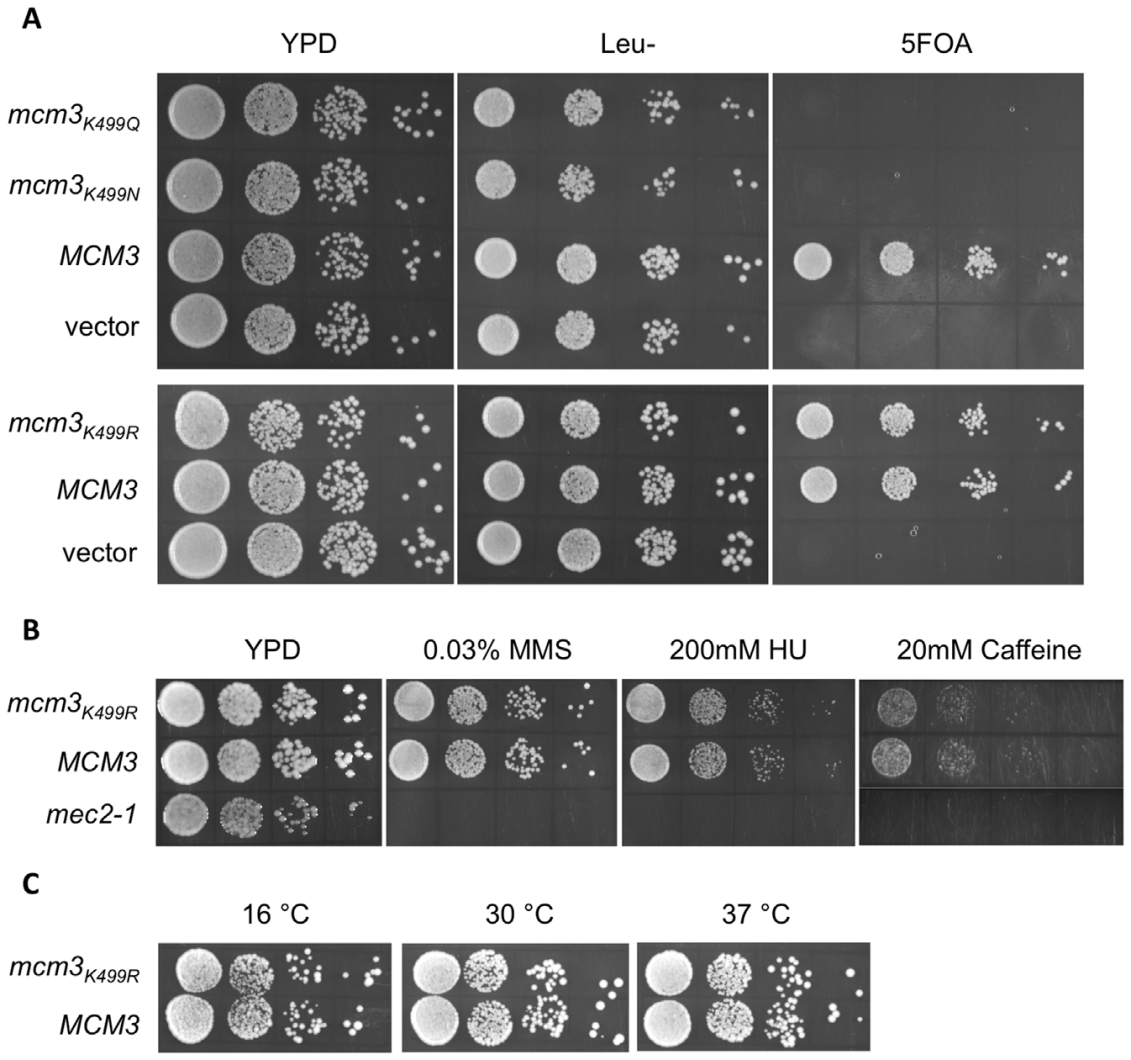
Characterization of *mcm3_K499R_*, *mcm3_K499N_*, and *mcm3_K499Q_* alleles. (A) The *MCM3*, *mcm3_K499R_*, *mcm3_K499N_*, and *mcm3_K499Q_* genes encoded on *LEU2*-containing centromeric plasmids, or empty plasmid (vector), were transformed into MDY16 (*mcm3*Δ YCplac33-*MCM3*). The transformed yeast were grown overnight at 30°C in liquid YPD, serially diluted, and spotted onto YPD, synthetic complete lacking leucine, and YPD containing 5-FOA. (B) The *MCM3* and *mcm3_K499R_* plasmid-shuffled strains were grown overnight at 30°C, serially diluted and spotted on YPD and YPD containing 0.03% MMS, 200 mM hydroxyurea (HU), or 20 mM caffeine. The *mec2-1* strain was subjected to the same growth assay as a positive control for genotoxic stress [51]. (C) The *MCM3* and *mcm3_K499R_* plasmid-shuffled strains were grown overnight at 30°C, serially diluted, spotted on YPD, and grown at 16°C, 30°C or 37°C.

The lysine residue on the PS1 hairpin is predicted to make an electrostatic interaction with the sugar phosphate backbone of DNA to facilitate translocation of DNA and unwinding [8]. We converted the lysine of the PS1 hairpin of Mcm3 to arginine, glutamine, or asparagine to determine whether the charge is important for function. Of the three alleles examined by plasmid shuffling, only *mcm3_K499R_* supported viability (Figure 5A). The strain containing this allele displayed no overt growth defects when plated on 200 mM hydroxyurea, 0.03% MMS, and 20 mM caffeine (Figure 5B). Additionally, the rate of growth was the same as wild type at 16°C, 30°C and 37°C (Figure 5C). This suggests that the positive charge at residue 499 of Mcm3 is essential for function.

**Figure 6:**
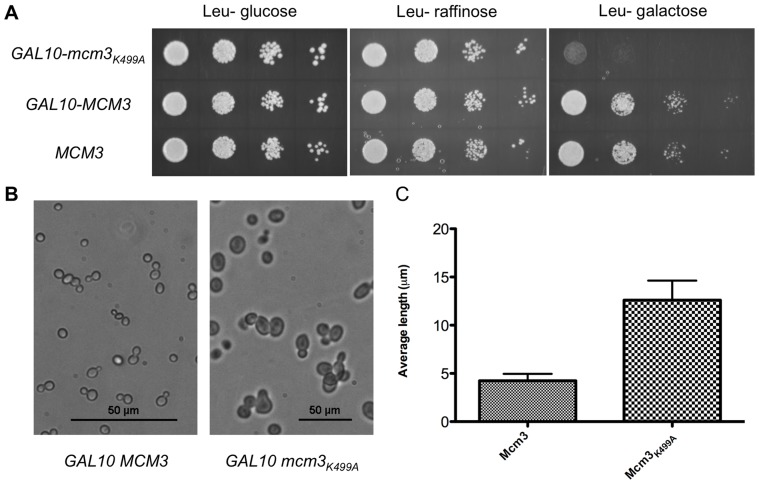
Effect of over-expressing the Mcm3_K499A_ subunit in a wild-type background. (A) BY4741 transformed with YCplac111-*GAL10*-*MCM3,* YCplac111-*GAL10-mcm3_K499A_*, or YCplac111-*GAL10* were grown overnight in media with 2% raffinose lacking leucine, serially diluted, and spotted onto plates lacking leucine and containing 2% glucose, 2% raffinose, or 2% galactose. (B) BY4741 bearing YCplac111-*GAL10-MCM3* or YCplac111-*GAL10-mcm3_K499A_* was grown in 2% galactose media lacking leucine and imaged using a Nikon Eclipse Ti microscope. Scale bars represent 50 µm. (C) The minimal diameter at the midsection was determined for BY4741 transformed with YCplac111-*GAL10-MCM3* or YCplac111-*GAL10*-*mcm3_K499A_* grown as above. The average diameter for 20 cells for each strain is shown with the standard deviation indicated.

To begin to investigate how Mcm3_K499A_ disrupts function, we addressed whether its overexpression would have a dominant negative effect. A plasmid expressing *mcm3_K499A_* or *MCM3* from a *GAL10* promoter was transformed into BY4741 (*MCM3*), and serial dilutions plated onto media containing glucose, raffinose, or galactose. In the presence of glucose or raffinose, where the *GAL10* promoter is transcriptionally repressed or not induced respectively, there was no effect on growth, whereas in galactose-containing media induction of *mcm3_K499A_* expression resulted in a slow growth phenotype (Figure 6A). In addition, there was an increase of approximately three-fold in cell diameter for the *GAL10-mcm3_K499A_* transformed strain compared to *GAL10-MCM3* transformed strain when grown in galactose-containing media (Figure 6B and 6C). Based on these observations, overexpression of *mcm3_K499A_* in the context of a wild type background leads to a dominant negative effect that is likely associated with a cell cycle defect.

### The Mcm3 PS1 hairpin is required for DNA unwinding

**Figure 7:**
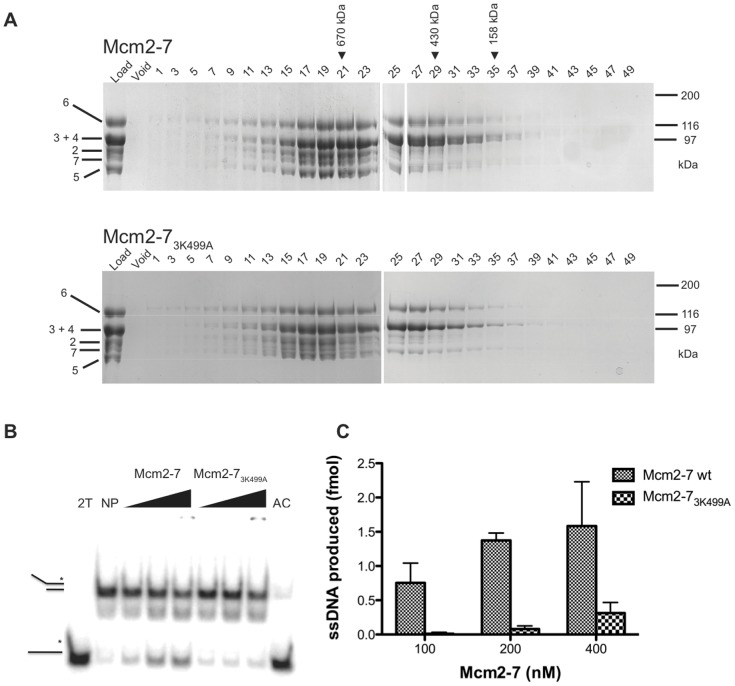
Reconstitution and analysis of Mcm2-7_3K499A_. (A) Individual Mcm subunits were expressed in bacteria, purified, and mixed in an equimolar ratio to reconstitute the hexameric complex. The Mcm2-7 complexes were then subjected to gel filtration chromatography, and fractions analyzed by SDS-PAGE. The top profile shows the wild-type Mcm2-7 complex, while the lower profile is the complex reconstituted with Mcm3_K499A_. (B) A synthetic forked substrate radiolabeled on the 5′ end (indicated by the asterisk) was incubated with 100, 200, and 400 nM of either Mcm2-7 or Mcm2-7_3K499A_ to assess the helicase activity of the reconstituted hexamers. The “no protein” (NP) lane indicates the position of the radiolabeled substrate DNA, and a sample containing only radiolabeled single strand DNA (2T) was used to mark the location of the liberated single strand product. The inability of the two single strands to re-anneal is demonstrated in the last lane (AC); here, the complementary (non radiolabeled) strand was added to the radiolabeled (2T) strand at the start of the helicase assay. (C) The relative ability of Mcm2-7 and Mcm2-7_3K499A_ to unwind DNA was quantitated by densitometric analysis of three replicates of the experiment shown in Panel B.

To determine the biochemical effects of Mcm3_K499A_ on the activity of the complex, we reconstituted it into Mcm2-7 to yield Mcm2-7_3K499A_. Each of the subunits, including Mcm3_K499A_ was expressed as a recombinant protein in *E. coli*, and checked for the absence of contaminating nuclease or ATPase activity. Individual Mcm subunits were mixed in equal molar ratios to reconstitute the hexameric complex, the final step of the reconstitution being elution from a gel filtration column. Mcm2-7_3K499A_ eluted at a volume corresponding to the MCM hexamer, similar to wild-type Mcm2-7 (∼600 kDa; Figure 7A). We examined the DNA unwinding of wild type and mutant Mcm complexes using a radiolabeled synthetic fork substrate where DNA unwinding is measured as the amount of single stranded DNA liberated from the duplex substrate. At a concentration of 200 nM the wild-type complex converted 1.5 fmoles of substrate to single-stranded DNA in 10 minutes (Figures 7B and 7C). By contrast, the 200 nM concentration of Mcm2-7_3K499A_ unwound 0.1 fmol of the fork substrate. Therefore Mcm2-7_3K499A_ has a ∼15-fold reduction in helicase activity, indicating that the Mcm3 PS1 hairpin is critical for DNA unwinding.

**Figure 8:**
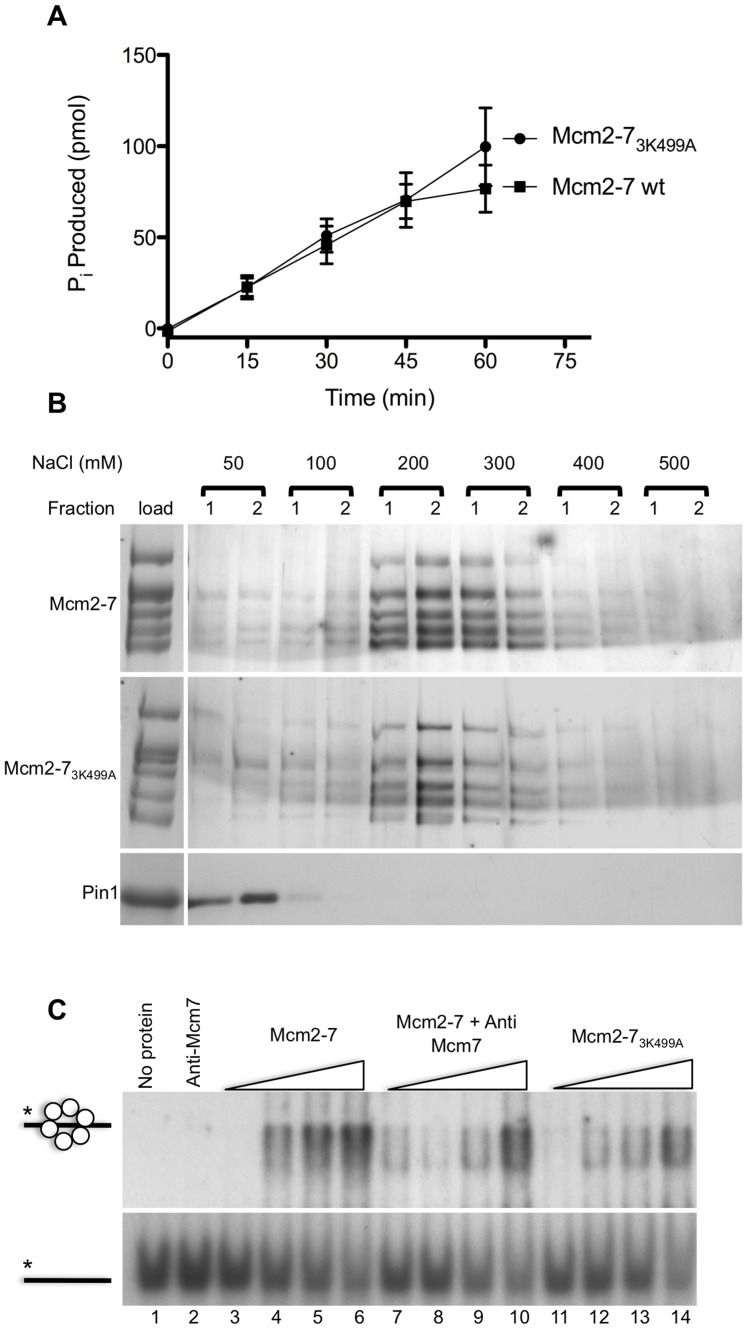
ATPase activity and DNA binding by Mcm2-7_3K499A_. (A) The reconstituted Mcm2-7 and Mcm2-7_3K499A_ complexes were tested for ATPase activity by incubating with [γ^32^P]-ATP and measuring the radioactivity of free P_i_ produced at the time points indicated. (B) Elution profile of Mcm2-7 and Mcm2-7_3K499A_ from ssDNA-Sepharose. Five micrograms of Mcm2-7 (upper panel), Mcm2-7_3K499A_ (middle panel), and the peptidyl prolyl isomerase Pin-1 (lower panel) were chromatographed on a 200 µL single-stranded DNA Sepharose column in the presence of 5 mM ATP. At each step the column was washed twice with 200 µL of buffer containing 50 mM, 100 mM, 200 mM, 300 mM, 400 mM, or 500 mM NaCl. Twenty-four microliters of each fraction was separated by SDS-PAGE (6%) and stained with colloidal blue to detect the load (left) and then silver stain to detect the fractions (right). (C) An electrophoretic mobility shift assay of DNA binding by Mcm2-7 (lanes 3-6) and Mcm2-7_3K499A_ (lanes 11–14). 5′ radiolabeled ATGTCCTAGCAAGCCAGAATTCGGCAGCGTC(T)_60_ was incubated with increasing concentrations (50, 100, 200, or 400 nM) of the Mcm hexameric complexes and separated in a 5% native polyacrylamide gel. Lanes 7-10 included a pre-incubation of the wild-type complex with an anti-Mcm7 antibody. Lanes 1 and 2 show the position of the DNA in the absence of protein, and in the presence of the anti-Mcm7 antibody, respectively.

The loss of helicase activity in Mcm2-7_3K499A_ may be due to a role for the Mcm3 PS1 hairpin in the ATPase activity of the complex. Interestingly, of the isolated dimer pairs, the pair of Mcm3 and Mcm7 has the highest ATPase activity, approaching that of the intact Mcm2-7 hexamer [25]. ATP hydrolysis was measured for intact wild-type Mcm2-7 and Mcm2-7_3K499A_ complexes. As shown in Figure 8A, the ATP hydrolysis rate for Mcm2-7_3K499A_ was not significantly different from the wild-type Mcm2-7. We next addressed whether Mcm2-7_3K499A_ is capable of single-stranded DNA binding. Mutant and wild-type complexes were chromatographed on a single-stranded Sepharose affinity column in the presence of ATP, and eluted with increasing salt concentration. As shown in Figure 8B, wild type Mcm2-7 eluted from this column primarily in the 200 and 300 mM NaCl wash fractions (upper panel). The elution profile for Mcm2-7_3K499A_ closely resembled that of the wild type complex (middle panel) indicating that the Mcm2-7_3K499A_ is capable of binding single stranded DNA. Lack of binding by the peptidyl prolyl isomerase Pin-1 (lower panel), a relatively basic protein with a pI of 9.4, indicated that the binding by the Mcm complexes was specific for DNA and not simply due to charge interactions. We next used an electrophoretic mobility shift assay in an attempt to detect more subtle differences in DNA binding. A 5′ radiolabeled oligonucleotide of 90 bases was used as the substrate. As shown in Figure 8C (lanes 3–6) increasing concentrations of wild-type Mcm2-7 depleted the substrate band and resulted in the appearance of a discrete band of reduced mobility. To confirm that the band of slower mobility was a Mcm2-7-DNA complex, Mcm7 antibody was pre-incubated with Mcm2-7 prior to addition of radiolabeled oligonucleotide (Figure 8C lanes 7-10). In the presence of the antibody the band of slower mobility was diminished, indicating that it contained Mcm2-7. When the DNA binding activity of Mcm2-7_3K499A_ was assayed (lanes 11–14), the amount of Mcm2-7_3K499A_-DNA complex was reduced suggesting that Mcm2-7_3K499A_ is less able to bind the 90 base single-stranded DNA or that the binding is unstable under the electrophoresis conditions.

**Figure 9:**
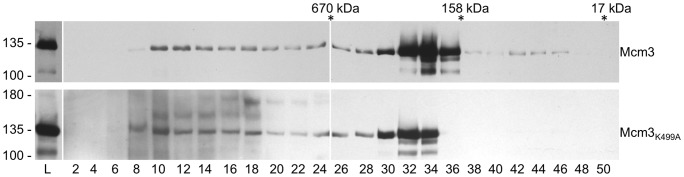
Analysis of myc^9^-tagged Mcm3 and Mcm3_K499A_ by gel filtration. Extracts were prepared from yeast strains MDY405 (myc^9^-Mcm3) and MDY406 (myc^9^-Mcm3_K499A_). Five mg of protein was separated on a Superose 6HR10/30 column. Protein from 10 µL aliquots of 250 µL fractions for Mcm3 and 20 µL for Mcm3_K449A_ were resolved by SDS-PAGE and myc-tagged protein detected by western blotting with anti-myc antibody. Fraction numbers are indicated below (L, load). The migration of molecular mass standards on the gel is shown on the left. The fractions corresponding to the peak elution of mass standards from the Superose 6 column is shown above.

The loss of helicase activity is the most pronounced functional effect of the Mcm3 hairpin mutation, and may explain the inviability of the *mcm3_K499A_* strain. To investigate the effects of the Mcm3 hairpin mutation in cells, we analyzed myc^9^-Mcm3_K499A_ expressed in yeast to determine if its ability to associate with other components required for replication differs from the wild type protein. Whole cell extracts containing myc^9^-tagged Mcm3 or Mcm3_K499A_ were prepared from cells grown to mid-log phase in YPD media and analyzed by gel filtration chromatography on a Superose 6 column. As shown in Figure 9, wild type Mcm3 elutes from the Superose 6 column in two peaks. The first peak is broad and corresponds to complexes with a molecular mass greater than 2 MDa. We suspect that this may represent the Mcm2-7 complex associated with chromatin. The second peak elutes in the molecular mass range from 150 to 350 kDa. This likely represents Mcm3 in association with other molecules, and perhaps an equilibrium between subcomplexes of Mcms. The elution profile of myc^9^-Mcm3_K499A_ resembled that of the wild type in that it eluted as two peaks, but with significant differences for the high- and low- molecular weight complexes. In the high molecular weight complex, Mcm3 appears as a single band that migrates with a mass of 135 kDa, while in the *mcm3_K499A_* strain, Mcm3 appears as a 135 kDa band but there are also two prominent bands at approximately 150 kDa and 175 kDa. These were not detected in the wild-type Mcm3 cells, even after prolonged exposure of the film. The reduced mobility forms of Mcm3 are likely the result of protein modification, but the nature of this modification is unclear. The second difference between wild-type and *mcm3_K499A_* cells is that the smaller complex from wild-type cells elutes with a peak at 200 kDa (fraction 34 on the profile, Figure 9), which was shifted to approximately 260 kDa in the *mcm3_K499A_* strain (fraction 32). Since the purified Mcm2-7_3K499A_ hexamer assembles and behaves similarly to the wild-type complex *in vitro,* these results suggest that in cells the altered activities of the Mcm2-7_3K499A_ complex result in changes in its molecular associations. Mcm3 has a unique role in the initiation process in that it is able to recruit its neighboring subunits, Mcm5 and Mcm7, to the origin recognition complex (ORC) independent of Cdt1 [44]. A winged helix domain only present at the C-terminal of Mcm3 interacts with the Cdc6/ORC complex to stimulate the ATP hydrolysis required for time-dependent stable double hexamer loading [45], [46]. It may be that these processes are compromised by the poor helicase activity of Mcm3_K499A_, leading to the differences we observe in Mcm3_K499A_-containing complexes.

**Figure 10:**
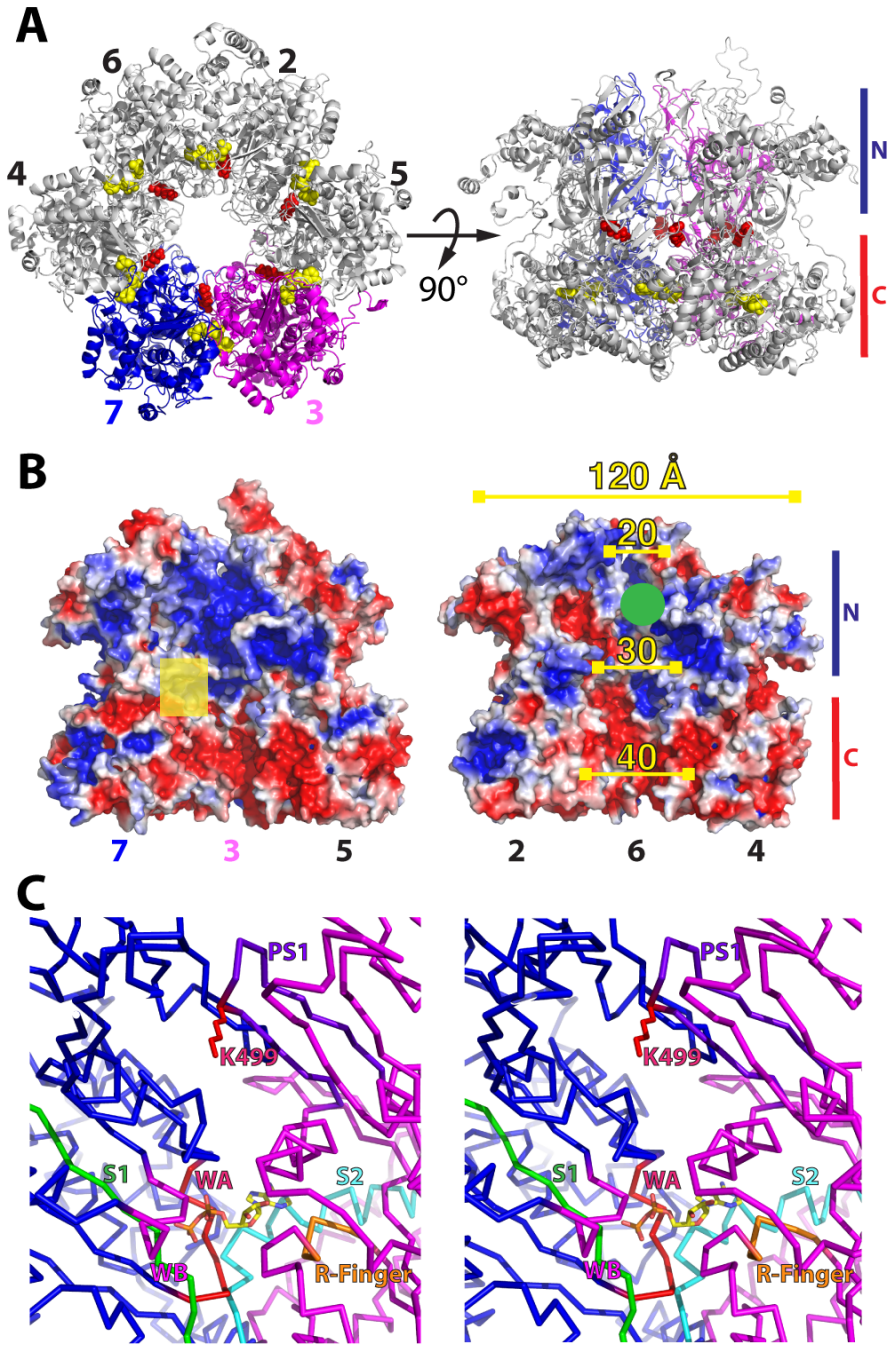
Model of Mcm2-7. (A) Two views of the Mcm2-7 hexamer: on the left, looking through the central channel from the C-terminal end, and on the right, rotated 90° about a horizontal axis. The N- and C-terminal domains of the subunits are indicated. Subunits 3 and 7 are highlighted in magenta and blue, respectively, and the side chains of the PS1 lysyl residues are shown as space-filling models in red. ADP modeled onto the Walker A motifs is shown as a yellow space-filling model. (B) The hexamer is split into two halves - Mcm7/3/5 and Mcm2/6/4 - to show the electrostatic surface of the Mcm2-7 central channel; red indicates a negative surface potential, and blue a positive surface potential. The outer diameter of the hexamer is approximately 120 Å, while the inner diameter decreases from approximately 40 Å in the region formed by the C-terminal domains, to 20 Å or less in the region formed by the N-terminal domains. The N-terminal region will be further constricted by a 70-residue insertion in Mcm6, which is expected to occupy the region indicated with a green circle. A yellow square on the Mcm7/3/5 trimer indicates the potential side exit tunnel in the Mcm3/7 interface, which is shown in greater detail in Panel C. (C) Stereodiagram of the potential exit channel in the Mcm3/7 interface, illustrating the position of the Mcm3 PS1 hairpin (purple) and essential K499 residue (red) relative to ADP bound to Mcm7 and the motifs associated with ATP binding and hydrolysis: the Walker A (WA, red), Walker B (WB, magenta), and Sensor 1 (S1, green) of Mcm7, and the Arginine Finger (orange) and Sensor 2 (S2, cyan) of Mcm3.

The observation that the Mcm3 PS1 hairpin is required for viability, while the PS1 hairpins of the other five subunits are individually dispensable provides insight into possible mechanisms for DNA unwinding by Mcm2-7. To understand the role of the Mcm3 PS1 hairpin, it is necessary to place it in the context of the Mcm2-7 hexamer, and to this end we modeled the structure of the Mcm2-7 complex using the structure of almost full-length SsoMCM monomer as a template for the individual Mcm2 through Mcm7 subunits; the hexameric structure of the N-terminal domain of *Methanobacterium thermoautotrophicum* MCM (mtMCM) was then used to assemble each of Mcm 2 through 7 into the hexameric Mcm2-7 complex (Figure 10A and 10B). Two interesting observations emerge from the Mcm2-7 model. First, the central channel exhibits a funnel-like shape, with the large opening formed by the C-terminal domains of the six subunits, and the smaller opening formed by the N-terminal domains. In fact, an extra domain in Mcm6 will further constrict the smaller N-terminal end of the Mcm2-7 hexamer. The Mcm6 domain comprises residues 407 to 475 and is inserted into a loop between two conserved β-strands in the SsoMCM and mtMCM structures; therefore its position inside the central channel is almost certain, although the degree to which it obstructs the central channel will depend on its structure, which has not been modeled. In addition to the funnel shape, the surface charge of the central channel exhibits a systematic change: the large opening formed by the C-terminal domains carries a negative surface charge, while the surface formed by the more constricted N-terminal domains is positively charged (Figure 10B).

The second important observation from the Mcm2-7 model is that the PS1 hairpins of all Mcm subunits are somewhat recessed and do not project into the interior of the central channel. Together with the reduced stability of DNA binding by Mcm2-7_3K499A_, these observations lead us to propose that the PS1 hairpin may be a component of an exit channel that directs one strand of incoming duplex DNA through the side of the Mcm2-7 hexamer. A structural model of the SsoMCM hexamer suggests that side channels are formed at the interface of subunits and run from the central channel to the outside of the ring [7]; the side channels are wide enough to accommodate single-stranded DNA. Similar channels are seen in electron micrographs of eukaryotic Mcm2-7 [47] and are present in our Mcm2-7 model (Figure 10C). The extrusion of single-stranded DNA through a side channel of the Mcm2-7 complex is a possible explanation for reduced helicase activity upon mutation of the Mcm3 PS1 hairpin. Of note, the channel incorporating the Mcm3 PS1 hairpin is formed at the interface between Mcm3 and Mcm7 (Figure 10C), which is critically important for Mcm2-7 function. For example, when the various Mcm subunits are expressed independently to generate dimeric species, it is the isolated Mcm3/7 dimer that has the highest ATPase activity, almost as high as the ATPase of the intact Mcm2-7 hexamer [26], [27]. Furthermore, expression of Mcm7 with mutations in its Walker A or Walker B motif, or expression of Mcm3 with an R542A mutation in its “arginine finger” lead to a strong dominant-lethal phenotype [26]. Taken together, the shape and charge features of the Mcm2-7 “funnel”, along with the functional importance of catalytic components found in the Mcm3/7 interface, are consistent with either double-stranded DNA or single-stranded DNA entering Mcm2-7 at the larger C-terminal end. If double-stranded DNA enters the channel, it may be destabilized due to the negative surface charge of the channel interior: one of the separated strands would be actively extruded through the Mcm3/7 interface, while the other strand could exit through the positively charged N-terminal end of the Mcm2-7 hexamer, or through another side channel. In a second possible scenario, single-stranded DNA would enter the Mcm2-7 channel and would be actively extruded through the Mcm3/7 interface, while the other strand would be sterically excluded from entering the channel.

The importance of the PS1 hairpins in Mcm function is most apparent from the loss of viability when PS1 of Mcm3 was mutated. We demonstrate that the Mcm3 PS1 hairpin participates in DNA unwinding by Mcm2-7, and based upon our *in vitro* experiments suggest that it may do so by altering the interaction of the complex with single-stranded DNA. This result is similar to findings with SsoMCM and the SF3 viral replicative helicases where the PS1 hairpin is essential for helicase activity [8], [9], [48]. We also note that the interactions of Mcm3_K499A_ are altered in cells as demonstrated by changes in its elution from a Superose 6 column. Whether these changes are due to or the cause of the defects in cellular function of the protein is unclear.

A key finding of our study is that of the six PS1 hairpins in the heterohexameric Mcm2-7 complex, only the PS1 hairpin of Mcm3 is essential. This strongly suggests that it has a unique role in Mcm2-7 function. The finding that the PS1 hairpin of Mcm3 is essential for viability is somewhat surprising since Mcm3 has been proposed to act principally in the regulation of the other Mcm2-7 subunits rather than have a direct role in DNA unwinding [49], [50]. Although not essential, the Mcm2, Mcm4, Mcm5, Mcm6 and Mcm7 PS1 hairpins are important for function as lysine to alanine mutations in any two subunits leads to inviability or slow growth. The finding that the *mcm4_K658A_ mcm5_K506A_* double mutation strain was viable, in contrast to the lack of viability of other pairwise combinations is also another clear indication that each subunit contributes differently to the function of Mcm2-7.

## Supporting Information

Figure S1 **Growth of** ***mcm3_K499A_*** **and** ***mcm7_K550A_*** **plasmid shuffled** **yeast strains.** Haploid yeast strains deleted for *MCM3* or *MCM7* and bearing *MCM3* or *MCM7* on a *URA3*-*CEN* plasmid were transformed with *LEU2-CEN* plasmids containing either *MCM3, mcm3_K499A_*, *MCM7*, *mcm7_K550A_* or the empty *LEU2-CEN* plasmid (Vector). The transformed yeast were grown overnight at 30°C in YPD media, serially diluted, and then spotted onto a YPD plate or a plate containing 5-FOA. The plates were incubated at 30°C for the number of days indicated.
(TIF)

Figure S2 **Expression levels of Mcm 2 and 3.** Yeast strains MDY70 (*MCM2*), MDY71 (*mcm2_K633A_*), MDY405 (*DED1-myc^9^-MCM3*) and MDY406 (*DED1-myc^9^-mcm3_K499A_*) were grown to mid-log phase; yeast extracts were prepared by grinding with glass beads, and 10, 20 or 40 µg of total protein separated by SDS-PAGE. Blots of these gels were probed with anti-Mcm2, (Santa Cruz Biotech) or anti-myc (Sigma-Aldrich) antibody to assess the level of Mcm subunit. We note that for Mcm3 detection the plasmids were transformed into BY4741 and thus contain wild-type Mcm3.
(TIF)

## References

[1] Bell SP, Dutta A (2002) DNA replication in eukaryotic cells. Annu Rev Biochem 71: 333–374. 12045100 10.1146/annurev.biochem.71.110601.135425

[2] Patel SS, Picha KM (2000) Structure and function of hexameric helicases. Annu Rev Biochem 69: 651–697. 10966472 10.1146/annurev.biochem.69.1.651

[3] Singleton MR, Dillingham MS, Wigley DB (2007) Structure and Mechanism of Helicases and Nucleic Acid Translocases. Annual Review of Biochemistry 76: 23–50. 10.1146/annurev.biochem.76.052305.115300 17506634

[4] Enemark EJ, Joshua-Tor L (2008) On helicases and other motor proteins. Curr Opin Struct Biol 18: 243–257. 18329872 10.1016/j.sbi.2008.01.007 PMC2396192

[5] Brewster AS, Chen XS (2010) Insights into the MCM functional mechanism: lessons learned from the archaeal MCM complex. Crit Rev Biochem Mol Biol 45: 243–256. 20441442 10.3109/10409238.2010.484836 PMC2953368

[6] Kaplan DL, Davey MJ, O'Donnell M (2003) Mcm4,6,7 uses a "pump in ring" mechanism to unwind DNA by steric exclusion and actively translocate along a duplex. J Biol Chem 278: 49171–49182. 13679365 10.1074/jbc.M308074200

[7] Brewster AS, Wang G, Yu X, Greenleaf WB, Carazo JM, et al (2008) Crystal structure of a near-full-length archaeal MCM: functional insights for an AAA+ hexameric helicase. Proc Natl Acad Sci U S A 105: 20191–20196. 19073923 10.1073/pnas.0808037105 PMC2629282

[8] Enemark EJ, Joshua-Tor L (2006) Mechanism of DNA translocation in a replicative hexameric helicase. Nature 442: 270–275. 16855583 10.1038/nature04943

[9] Gai D, Zhao R, Li D, Finkielstein CV, Chen XS (2004) Mechanisms of conformational change for a replicative hexameric helicase of SV40 large tumor antigen. Cell 119: 47–60. 15454080 10.1016/j.cell.2004.09.017

[10] Bae B, Chen YH, Costa A, Onesti S, Brunzelle JS, et al (2009) Insights into the architecture of the replicative helicase from the structure of an archaeal MCM homolog. Structure 17: 211–222. 19217392 10.1016/j.str.2008.11.010

[11] Enemark EJ, Joshua-Tor L (2006) Mechanism of DNA translocation in a replicative hexameric helicase. Nature 442: 270–275. 16855583 10.1038/nature04943

[12] McGeoch AT, Trakselis MA, Laskey RA, Bell SD (2005) Organization of the archaeal MCM complex on DNA and implications for the helicase mechanism. Nat Struct Mol Biol 12: 756–762. 16116441 10.1038/nsmb974

[13] Bochman ML, Schwacha A (2009) The Mcm complex: unwinding the mechanism of a replicative helicase. Microbiol Mol Biol Rev 73: 652–683. 19946136 10.1128/MMBR.00019-09 PMC2786579

[14] Forsburg SL (2004) Eukaryotic MCM proteins: beyond replication initiation. Microbiol Mol Biol Rev 68: 109–131. 15007098 10.1128/MMBR.68.1.109-131.2004 PMC362110

[15] Brown GW, Kelly TJ (1998) Purification of Hsk1, a minichromosome maintenance protein kinase from fission yeast. J Biol Chem 273: 22083–22090. 9705352 10.1074/jbc.273.34.22083

[16] Bruck I, Kaplan D (2009) Dbf4-Cdc7 phosphorylation of Mcm2 is required for cell growth. J Biol Chem 284: 28823–28831. 19692334 10.1074/jbc.M109.039123 PMC2781428

[17] Charych DH, Coyne M, Yabannavar A, Narberes J, Chow S, et al (2008) Inhibition of Cdc7/Dbf4 kinase activity affects specific phosphorylation sites on MCM2 in cancer cells. J Cell Biochem 104: 1075–1086. 18286467 10.1002/jcb.21698

[18] Chuang LC, Teixeira LK, Wohlschlegel JA, Henze M, Yates JR, et al (2009) Phosphorylation of Mcm2 by Cdc7 promotes pre-replication complex assembly during cell-cycle re-entry. Mol Cell 35: 206–216. 19647517 10.1016/j.molcel.2009.06.014 PMC2725784

[19] Lei M, Kawasaki Y, Young MR, Kihara M, Sugino A, et al (1997) Mcm2 is a target of regulation by Cdc7-Dbf4 during the initiation of DNA synthesis. Genes Dev 11: 3365–3374. 9407029 10.1101/gad.11.24.3365 PMC316824

[20] Masai H, Taniyama C, Ogino K, Matsui E, Kakusho N, et al (2006) Phosphorylation of MCM4 by Cdc7 kinase facilitates its interaction with Cdc45 on the chromatin. J Biol Chem 281: 39249–39261. 17046832 10.1074/jbc.M608935200

[21] Montagnoli A, Valsasina B, Brotherton D, Troiani S, Rainoldi S, et al (2006) Identification of Mcm2 phosphorylation sites by S-phase-regulating kinases. J Biol Chem 281: 10281–10290. 16446360 10.1074/jbc.M512921200

[22] Sheu YJ, Stillman B (2006) Cdc7-Dbf4 phosphorylates MCM proteins via a docking site-mediated mechanism to promote S phase progression. Mol Cell 24: 101–113. 17018296 10.1016/j.molcel.2006.07.033 PMC2923825

[23] Sheu YJ, Stillman B (2010) The Dbf4-Cdc7 kinase promotes S phase by alleviating an inhibitory activity in Mcm4. Nature 463: 113–117. 20054399 10.1038/nature08647 PMC2805463

[24] Tsuji T, Ficarro SB, Jiang W (2006) Essential role of phosphorylation of MCM2 by Cdc7/Dbf4 in the initiation of DNA replication in mammalian cells. Mol Biol Cell 17: 4459–4472. 16899510 10.1091/mbc.E06-03-0241 PMC1635350

[25] Stead BE, Brandl CJ, Davey MJ Phosphorylation of Mcm2 modulates Mcm2-7 activity and affects the cell's response to DNA damage. Nucleic Acids Res 39: 6998–7008. 21596784 10.1093/nar/gkr371 PMC3167627

[26] Bochman ML, Bell SP, Schwacha A (2008) Subunit organization of Mcm2-7 and the unequal role of active sites in ATP hydrolysis and viability. Mol Cell Biol 28: 5865–5873. 18662997 10.1128/MCB.00161-08 PMC2547011

[27] Davey MJ, Indiani C, O'Donnell M (2003) Reconstitution of the Mcm2–7p heterohexamer, subunit arrangement, and ATP site architecture. J Biol Chem 278: 4491–4499. 12480933 10.1074/jbc.M210511200

[28] Schwacha A, Bell SP (2001) Interactions between two catalytically distinct MCM subgroups are essential for coordinated ATP hydrolysis and DNA replication. Mol Cell 8: 1093–1104. 11741544 10.1016/s1097-2765(01)00389-6

[29] Forsburg SL, Sherman DA, Ottilie S, Yasuda JR, Hodson JA (1997) Mutational analysis of Cdc19p, a *Schizosaccharomyces pombe* MCM protein. Genetics 147: 1025–1041. 9383050 10.1093/genetics/147.3.1025 PMC1208231

[30] Bochman ML, Schwacha A (2008) The Mcm2-7 Complex Has In Vitro Helicase Activity. Molecular Cell 31: 287–293. 18657510 10.1016/j.molcel.2008.05.020

[31] Stead BE, Sorbara CD, Brandl CJ, Davey MJ (2009) ATP Binding and Hydrolysis by Mcm2 Regulate DNA Binding by Mcm Complexes. Journal of Molecular Biology 391: 301–313. 19540846 10.1016/j.jmb.2009.06.038 PMC5154746

[32] Gietz RD, Sugino A (1988) New yeast-*Escherichia coli* shuttle vectors constructed with in vitro mutagenized yeast genes lacking six-base pair restriction sites. Gene 74: 527–534. 3073106 10.1016/0378-1119(88)90185-0

[33] Hoke SM, Irina Mutiu A, Genereaux J, Kvas S, Buck M, et al. Mutational analysis of the C-terminal FATC domain of Saccharomyces cerevisiae Tra1. Curr Genet 56: 447–465. 20635087 10.1007/s00294-010-0313-3 PMC2943577

[34] Boeke JD, Trueheart J, Natsoulis G, Fink GR (1987) 5-Fluoroorotic acid as a selective agent in yeast molecular genetics. Methods Enzymol 154: 164–175. 3323810 10.1016/0076-6879(87)54076-9

[35] Scherer S, Davis RW (1979) Replacement of chromosome segments with altered DNA sequences constructed in vitro. Proc Natl Acad Sci U S A 76: 4951–4955. 388424 10.1073/pnas.76.10.4951 PMC413056

[36] Mutiu AI, Hoke SM, Genereaux J, Hannam C, MacKenzie K, et al (2007) Structure/function analysis of the phosphatidylinositol-3-kinase domain of yeast tra1. Genetics 177: 151–166. 17660562 10.1534/genetics.107.074476 PMC2013730

[37] Saleh A, Lang V, Cook R, Brandl CJ (1997) Identification of native complexes containing the yeast coactivator/repressor proteins NGG1/ADA3 and ADA2. J Biol Chem 272: 5571–5578. 9038164 10.1074/jbc.272.9.5571

[38] Chivian D, Baker D (2006) Homology modeling using parametric alignment ensemble generation with consensus and energy-based model selection. Nucleic Acids Res 34: e112. 16971460 10.1093/nar/gkl480 PMC1635247

[39] Raman S, Vernon R, Thompson J, Tyka M, Sadreyev R, et al (2009) Structure prediction for CASP8 with all-atom refinement using Rosetta. Proteins 77 Suppl 989–99. 19701941 10.1002/prot.22540 PMC3688471

[40] Fletcher RJ, Bishop BE, Leon RP, Sclafani RA, Ogata CM, et al (2003) The structure and function of MCM from archaeal M. Thermoautotrophicum. Nat Struct Biol 10: 160–167. 12548282 10.1038/nsb893

[41] Dolinsky TJ, Nielsen JE, McCammon JA, Baker NA (2004) PDB2PQR: an automated pipeline for the setup of Poisson-Boltzmann electrostatics calculations. Nucleic Acids Res 32: W665–667. 15215472 10.1093/nar/gkh381 PMC441519

[42] Baker NA, Sept D, Joseph S, Holst MJ, McCammon JA (2001) Electrostatics of nanosystems: application to microtubules and the ribosome. Proc Natl Acad Sci U S A 98: 10037–10041. 11517324 10.1073/pnas.181342398 PMC56910

[43] Maine GT, Sinha P, Tye BK (1984) Mutants of *S. cerevisiae* defective in the maintenance of minichromosomes. Genetics 106: 365–385. 6323245 10.1093/genetics/106.3.365 PMC1224244

[44] Moreau MJ, McGeoch AT, Lowe AR, Itzhaki LS, Bell SD (2007) ATPase site architecture and helicase mechanism of an archaeal MCM. Mol Cell 28: 304–314. 17964268 10.1016/j.molcel.2007.08.013

[45] Frigola J, Remus D, Mehanna A, Diffley JF ATPase-dependent quality control of DNA replication origin licensing. (2013). Nature 495: 339–343. 23474987 10.1038/nature11920 PMC4825857

[46] Fernandez-Cid A, Riera A, Tognetti S, Herrera MC, Samel S, et al An ORC/Cdc6/MCM2-7 complex is formed in a multistep reaction to serve as a platform for MCM double-hexamer assembly. (2013). Mol Cell 50: 577–588. 23603117 10.1016/j.molcel.2013.03.026

[47] Remus D, Beuron F, Tolun G, Griffith JD, Morris EP, et al (2009) Concerted loading of Mcm2-7 double hexamers around DNA during DNA replication origin licensing. Cell 139: 719–730. 19896182 10.1016/j.cell.2009.10.015 PMC2804858

[48] McGeoch AT, Trakselis MA, Laskey RA, Bell SD (2005) Organization of the archaeal MCM complex on DNA and implications for the helicase mechanism. Nat Struct Mol Biol 12: 756–762. 16116441 10.1038/nsmb974

[49] Lee JK, Hurwitz J (2000) Isolation and characterization of various complexes of the minichromosome maintenance proteins of *Schizosaccharomyces pombe* . J Biol Chem 275: 18871–18878. 10770926 10.1074/jbc.M001118200

[50] Tye BK, Sawyer S (2000) The Hexameric Eukaryotic MCM Helicase: Building Symmetry from Nonidentical Parts. J Biol Chem 275: 34833–34836. 10980206 10.1074/jbc.R000018200

[51] Weinert TA, Kiser GL, Hartwell LH (1994) Mitotic checkpoint genes in budding yeast and the dependence of mitosis on DNA replication and repair. Genes & Development 8: 652–665. 7926756 10.1101/gad.8.6.652

[52] Winzeler EA, Davis RW (1997) Functional analysis of the yeast genome. Curr Opin Genet Dev 7: 771–776. 9468786 10.1016/s0959-437x(97)80039-1

