# Supplementary figures and images for: The PS1 Hairpin of Mcm3 Is Essential for Viability and for DNA Unwinding In Vitro
Source: PLoS One. 2013 Dec 11;8(12):e82177. doi: 10.1371/journal.pone.0082177 (PMC3859580; doi:10.1371/journal.pone.0082177)

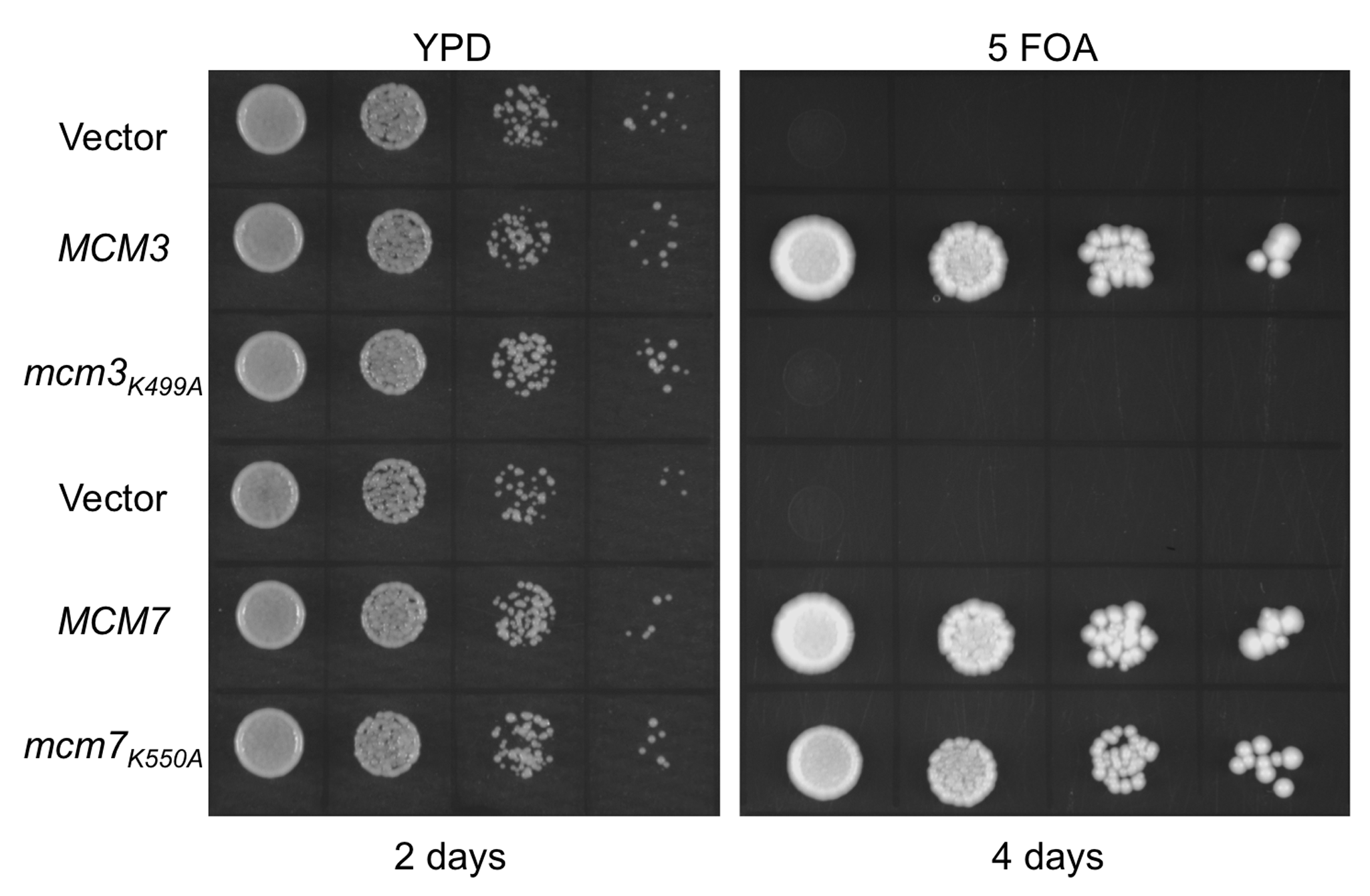

Supplement: Figure S1 — Growth of mcm3K499A and mcm7K550A plasmid shuffled yeast strains. Haploid yeast strains deleted for MCM3 or MCM7 and bearing MCM3 or MCM7 on a URA3-CEN plasmid were transformed with LEU2-CEN plasmids containing either MCM3, mcm3K499A, MCM7, mcm7K550A or the empty LEU2-CEN plasmid (Vector). The transformed yeast were grown overnight at 30°C in YPD media, serially diluted, and then spotted onto a YPD plate or a plate containing 5-FOA. The plates were incubated at 30°C for the number of days indicated. (TIF) [file pone.0082177.s001.tif]

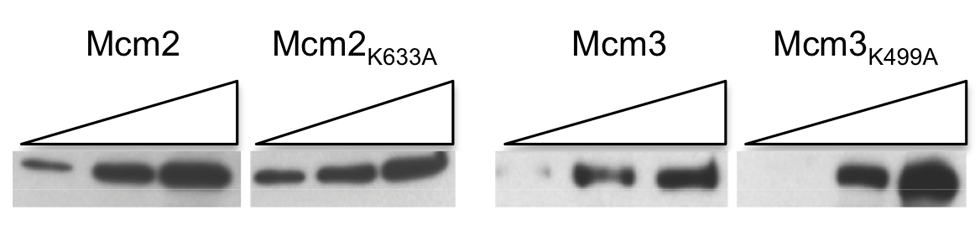

Supplement: Figure S2 — Expression levels of Mcm 2 and 3. Yeast strains MDY70 (MCM2), MDY71 (mcm2K633A), MDY405 (DED1-myc9-MCM3) and MDY406 (DED1-myc9-mcm3K499A) were grown to mid-log phase; yeast extracts were prepared by grinding with glass beads, and 10, 20 or 40 µg of total protein separated by SDS-PAGE. Blots of these gels were probed with anti-Mcm2, (Santa Cruz Biotech) or anti-myc (Sigma-Aldrich) antibody to assess the level of Mcm subunit. We note that for Mcm3 detection the plasmids were transformed into BY4741 and thus contain wild-type Mcm3. (TIF) [file pone.0082177.s002.tif]
